# *In Vitro* Profiling of Commonly Used Post-transplant Immunosuppressants Reveals Distinct Impact on Antiviral T-cell Immunity Towards CMV

**DOI:** 10.3389/ti.2024.12720

**Published:** 2024-04-09

**Authors:** Markus Benedikt Krueger, Agnes Bonifacius, Anna Christina Dragon, Maria Michela Santamorena, Björn Nashan, Richard Taubert, Ulrich Kalinke, Britta Maecker-Kolhoff, Rainer Blasczyk, Britta Eiz-Vesper

**Affiliations:** ^1^ Institute of Transfusion Medicine and Transplant Engineering, Hannover Medical School, Hannover, Germany; ^2^ German Center for Infection Research (DZIF), Braunschweig, Germany; ^3^ Clinic for Hepatopancreaticobiliary Surgery and Transplantation, First Affiliated Hospital, University of Science and Technology of China, Hefei, China; ^4^ Department of Gastroenterology, Hepatology, Infectious Diseases and Endocrinology, Hannover Medical School, Hannover, Germany; ^5^ TWINCORE, Centre for Experimental and Clinical Infection Research, A Joint Venture Between the Helmholtz Centre for Infection Research and the Hannover Medical School, Hannover, Germany; ^6^ Department of Pediatric Hematology and Oncology, Hannover Medical School, Hannover, Germany

**Keywords:** CMV-specific T cells, immunosuppression, adoptive T-cell therapy, solid organ transplantation, hematopoietic stem cell transplantation

## Abstract

Infectious complications, including widespread human cytomegalovirus (CMV) disease, frequently occur after hematopoietic stem cell and solid organ transplantation due to immunosuppressive treatment causing impairment of T-cell immunity. Therefore, in-depth analysis of the impact of immunosuppressants on antiviral T cells is needed. We analyzed the impact of mTOR inhibitors sirolimus (SIR/S) and everolimus (EVR/E), calcineurin inhibitor tacrolimus (TAC/T), purine synthesis inhibitor mycophenolic acid (MPA/M), glucocorticoid prednisolone (PRE/P) and common double (T+S/E/M/P) and triple (T+S/E/M+P) combinations on antiviral T-cell functionality. T-cell activation and effector molecule production upon antigenic stimulation was impaired in presence of T+P and triple combinations. SIR, EVR and MPA exclusively inhibited T-cell proliferation, TAC inhibited activation and cytokine production and PRE inhibited various aspects of T-cell functionality including cytotoxicity. This was reflected in an *in vitro* infection model, where elimination of CMV-infected human fibroblasts by CMV-specific T cells was reduced in presence of PRE and all triple combinations. CMV-specific memory T cells were inhibited by TAC and PRE, which was also reflected with double (T+P) and triple combinations. EBV- and SARS-CoV-2-specific T cells were similarly affected. These results highlight the need to optimize immune monitoring to identify patients who may benefit from individually tailored immunosuppression.

## Graphical Abstract

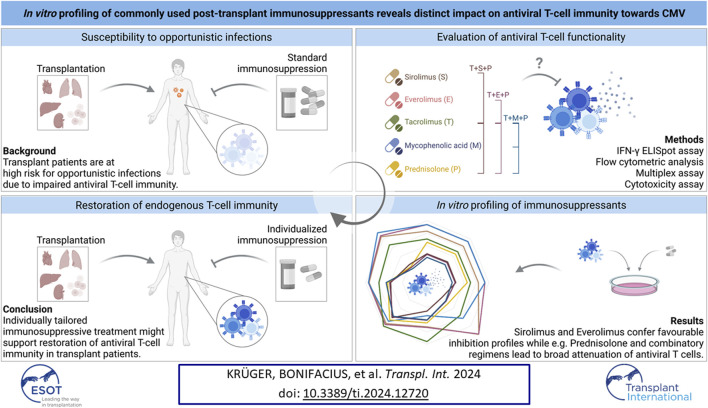

## Introduction

Infectious complications following hematopoietic stem cell and solid organ transplantation (HSCT, SOT) are common due to immunosuppressive treatment for prevention of graft-versus-host disease (GvHD) and allograft rejection. Persistent herpesviruses, such as human cytomegalovirus (CMV), are particularly frequent pathogens. An association between CMV infection/reactivation, the development and severity of GvHD and graft injury has been described in several clinical studies of HSCT and SOT [1–3]. Risk factors include *in vivo* or *in vitro* T-cell depletion, HLA-mismatched HSCT, the intensity of immunosuppression, and - in the setting of SOT - the type of transplanted organ [4, 5]. Moreover, CMV-seronegative (CMV-) SOT recipients of a graft from a CMV-seropositive (CMV+) donor (D+/R-) are at high-risk, with incidences of CMV disease up to 50% [6, 7].

The two main strategies to prevent CMV infection or disease in transplant patients are antiviral prophylaxis and preemptive therapy. Especially in high-risk SOT recipients, the most common strategy is antiviral prophylaxis, which is applied for up to 12 months after transplantation. Despite effectiveness of antiviral prophylaxis, side-effects such as nephrotoxicity or bone marrow suppression can result in discontinuation of prophylaxis and late-onset CMV disease after end of prophylaxis [8]. In addition, drug resistances can limit the efficacy of antiviral drugs [9–11]. In 2017/2018, letermovir was approved for prophylaxis after HSCT. In a recent phase III clinical trial comparing valganciclovir and letermovir prophylaxis in kidney transplant recipients (D+/R-), similar incidences of CMV disease were observed in both groups, with fewer side effects in patients receiving letermovir [12]. Preemptive treatment comprises of regular monitoring of viral load, allowing rapid therapy initiation upon detection of an increase. By this, progression to CMV disease can be prevented at an early stage of virus replication while at the same time, myelotoxicity associated with antiviral drugs is reduced [4, 13].

Mechanistically, a relationship between the magnitude of T-cell responses, especially by CD8^+^ T cells, CMV clearance and restoration of antiviral immunity was found [14]. In line, late-onset CMV disease and mortality have been correlated with the absence of CMV-specific T cells [7, 15, 16]. In recent studies, lower incidence of late-onset CMV disease was observed in liver transplant patients receiving preemptive therapy compared to prophylaxis and this was hypothesized to be due to enhanced CMV-specific T-cell immunity [17, 18]. Assuming that preemptive treatment potentially allows early immune reconstitution and the establishment of cellular antiviral immunity due to controlled low-level CMV replication, the restoration of endogenous antiviral immunity may be sensitively disrupted or delayed by immunosuppressive therapy.

Appropriate T-cell function relies on a variety of aspects and these are targeted via different mechanisms by post-transplant immunosuppressants. Reduction of immunosuppression as tolerated is an alternative option to restore a functional antiviral immune response. CMV disease after SOT typically occurs after 30–90 days [19–22]. At this point, patients are mostly treated by maintenance therapy, e.g., triple combinations usually consisting of a calcineurin inhibitor (CNI, e.g., tacrolimus) and a corticosteroid (e.g., prednisolone), supplemented with a purine synthesis inhibitor (e.g., mycophenolate mofetil, MMF) or a mechanistic target of rapamycin inhibitor (mTORi, e.g., sirolimus, everolimus). Of note, different clinical studies including the ATHENA study showed that the use of an mTORi was associated with lower CMV infection incidences compared to MMF-based regimens [23–29].

To support the restoration of antiviral immunity in SOT recipients and thereby reduce the risk of viral infection or reactivation, in-depth analysis of the effects of immunosuppressive drugs and combination regimens on antiviral T cells is required. In this study, we analyzed the impact of mTORi sirolimus (S/SIR) and everolimus (E/EVR), the CNI tacrolimus (T/TAC), the active metabolite of the purine synthesis inhibitor MMF - mycophenolic acid (M/MPA) - and the glucocorticoid prednisolone (P/PRE) [30] on CMV-specific T cells. As combination regimens are often used due to synergistic effects and lower single doses thereby minimizing toxicities, we included double (T+S/E/M/P) and triple combinations (T+S/E/M+P) in our study. Detailed assessment of CMV-specific T-cell responses *in vitro* revealed that SIR, EVR and MPA selectively inhibited T-cell proliferation, TAC slightly inhibited different aspects of CMV-specific T-cell functionality and PRE had broad inhibitory effects. Severe impairment was observed with triple combinations, and this could not be compensated by mTORi harboring partial beneficial effects on CMV-specific T cells. In line with that, T+P impaired antiviral T-cell functionality more strongly than T+S/E/M. These results, including evidence of a similar effect on T cells against EBV and SARS-CoV-2, highlight the need to optimize monitoring of immunocompromised patients or patients with viral infection/reactivation by determining antigen-specific T-cell functionality to further individualize immunosuppressive therapy.

## Materials and Methods

For description of methods please see Supplementary Material.

## Results

### PRE and Triple Combinations Reduce Antiviral T-cell Activation and Effector Molecule Production

**FIGURE 1:**
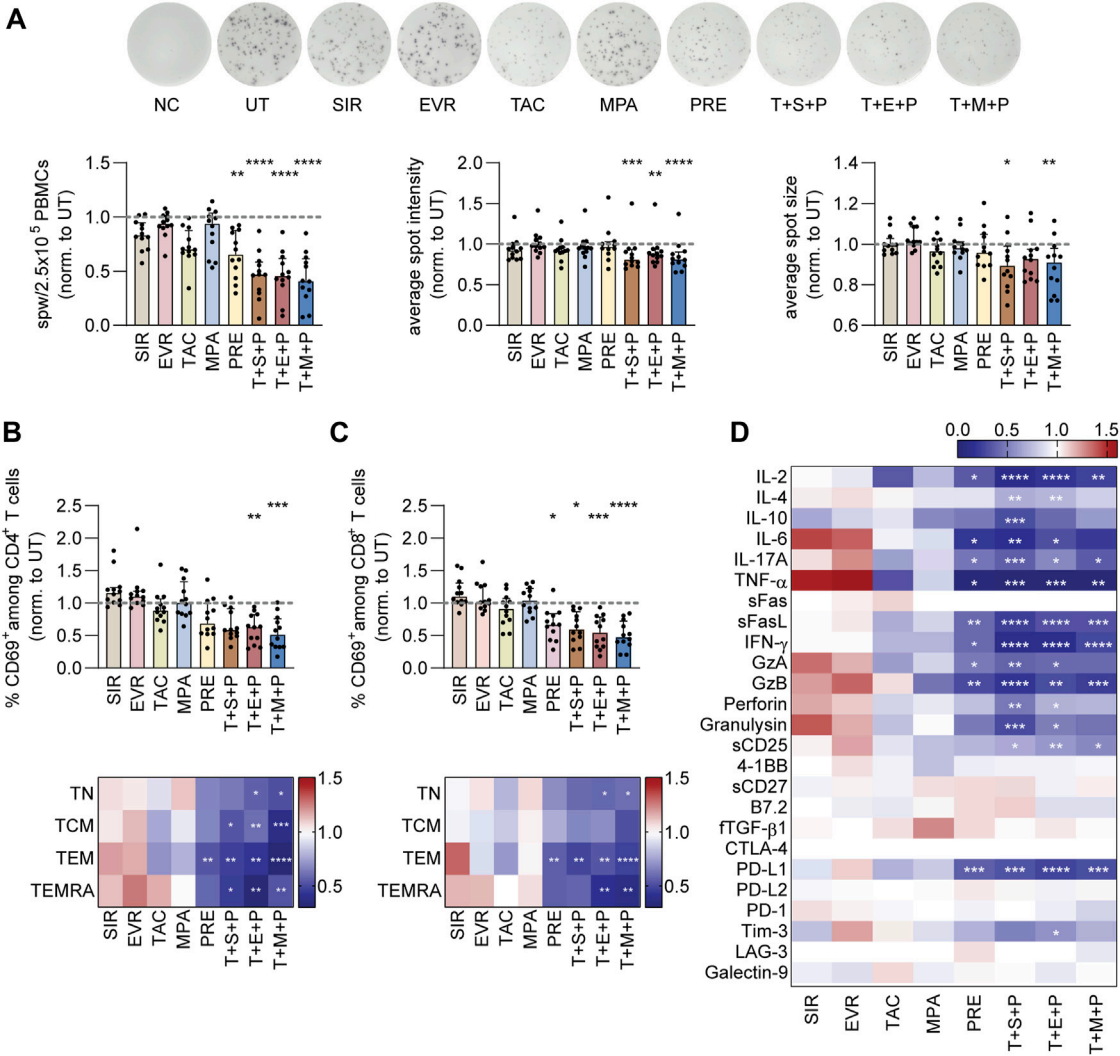
IFN-γ ELISpot, activation and cytokine secretion of CMV_pp65-stimulated PBMCs under immunosuppression. **(A)** PBMCs were isolated from CMV+ donors, rested overnight and stimulated with CMV_pp65 on day 1 in presence and absence of indicated immunosuppressants on IFN-γ ELISpot plates. After 24h, secreted IFN-γ was detected. Representative and summarized IFN-γ ELISpot results shown as spots per well (spw)/2.5 × 10^5^ PBMCs, spot intensity and spot size, normalized to untreated control (UT). **(B–D)** PBMCs were isolated from CMV+ donors, rested overnight and stimulated with CMV_pp65 on day 1 in presence and absence of indicated immunosuppressants. After 24 h cells were harvested for flow cytometric analysis and cell culture supernatants were collected for multiplex analysis. **(B)** Frequencies of CD69^+^ cells among CD4^+^ T cells (bar graph) and memory CD4^+^ T-cell subsets (heat map), normalized to UT. **(C)** Summarized frequencies of CD69^+^ cells among CD8^+^ T cells and memory CD8^+^ T-cell subsets (heat map), normalized to UT. **(B–C)** Bar graphs show median and interquartile range Q1-Q3, each symbol represents data from one donor (n = 12). **(B–D)** Heat maps show median values, normalized to UT (n = 12). Statistical significance (in comparison to UT) was calculated using **(A–C)** Friedman test followed by Dunn’s multiple comparison and **(D)** 2way ANOVA followed by Dunnett’s multiple comparison. *p < 0.05, **p < 0.01, ***p < 0.001, ****p < 0.0001. NC negative control (unstimulated), UT untreated, SIR/S sirolimus, EVR/E everolimus, TAC/T tacrolimus, MPA/M mycophenolic acid, PRE/P prednisolone, TN naïve T cells (CD45RA^+^/CD62L^+^), TCM central memory T cells (CD45RA^−^/CD62L^+^), TEM effector memory T cell (CD45RA^−^/CD62L^−^), TEMRA effector memory T cell re-expressing CD45RA (CD45RA^+^/CD62L^−^).

To analyze the impact of the different immunosuppressants on the reactivity of CMV-specific memory T cells, PBMCs were isolated from CMV+ healthy donors and subjected to IFN-γ ELISpot assay using CMV_pp65 overlapping peptide pool for restimulation in absence or presence of immunosuppressants (Figure 1A; Supplementary Figure S1A). To account for inter-individual differences (Supplementary Figure S1A), the data were normalized to values obtained from untreated (UT; stimulated but not treated with immunosuppressants) controls (Figure 1A). The frequencies of reactive CMV-specific T cells were significantly decreased upon treatment with PRE and T+S/E/M+P. SIR and TAC slightly reduced detectable CMV-specific T-cell response. In addition to the number of spots, correlating to the number of reactive CMV-specific memory T cells, average spot intensities and sizes were significantly reduced in presence of triple combinations. Since all triple combinations severely impaired memory T-cell reactivity, we analyzed the impact of double combination of immunosuppressants (T+S/E/P) on the reactivity of CMV-specific T cells in a small donor cohort, revealing significantly reduced number of spots in the presence of T+P (Supplementary Figure S1B). Of note, EBV- and SARS-CoV-2-specific T-cell responses were similarly affected by immunosuppressive treatment (Supplementary Figures S1C, S1D), with SARS-CoV-2-specific T cells being more susceptible.

To gain more insights into the affected T-cell populations, PBMCs from CMV+ donors were stimulated with CMV_pp65 for 24 h in absence or presence of immunosuppressants, followed by analysis of CD69 expression as indicator of activation (Figures 1B, C; Supplementary Figures S2A, S2B). Frequencies of CD69^+^ T cells after antigenic stimulation varied between donors and T-cell subsets (Fig. S2b) and were normalized to values obtained from UT controls (Figures 1B, C). Activation of CD4^+^ T cells by CMV_pp65 was significantly reduced in presence of T+E+P and T+M+P (Figure 1B). Of note, within the different CD4^+^ memory T-cell subsets, activation was significantly reduced in presence of all triple combinations. Moreover, in presence of PRE, CD4^+^ effector memory T cells (TEM, CD45RA^−^CD62L^−^) were significantly less activated. Slightly reduced CD69 expression on CD4^+^ central memory T cells (TCM, CD45RA^−^CD62L^+^) and TEM was detected in presence of TAC and MPA. Similarly, activation of CD8^+^ T cells by CMV_pp65 was significantly reduced in presence of triple combinations and PRE (Figure 1C). The main affected CD8^+^ memory T-cell subsets were TEM and effector memory T cells re-expressing CD45RA (TEMRA, CD45RA^+^CD62L^−^). In line with the effect of PRE on CD4^+^ T cells, significant reduction of CD69 expression among CD8^+^ TEM was observed in presence of PRE. Of note, slightly increased activation of CD4^+^ and CD8^+^ TEM and TEMRA were observed in presence of SIR and EVR. In a small donor cohort, T-cell activation was analyzed after antigenic restimulation in presence of double combinations of immunosuppressive drugs (T+S/E/M/P) and found to be slightly reduced in presence of T+P (Supplementary Figure S2C). Similar tendencies were observed for EBV- and SARS-CoV-2-specific T-cell responses (Supplementary Figures S2D, S2E). The activation of CD4^+^ and CD8^+^ SARS-CoV-2-specific T cells was significantly reduced in presence of T+P (Supplementary Figure S2E).

For a more comprehensive overview on the impact of immunosuppression on the production of cytotoxic mediators, multiplex cytokine assays were performed with supernatants of CMV_pp65-stimulated PBMCs (Figure 1D; Supplementary Figure S3). The raw values (Supplementary Figure S3) were normalized to the values obtained from UT controls (Figure 1D). While SIR and EVR induced slightly higher concentrations of, e.g., IL-6 and TNF-α, the secretion of pro-inflammatory effector molecules was slightly reduced in presence of TAC, MPA and significantly reduced in presence of PRE and T+S/E/M+P. To confirm antiviral T cells as source of the measured effector molecules, we analyzed the culture supernatants of T-cell-depleted PBMCs (Supplementary Figure S4A) stimulated with CMV_pp65 (Supplementary Figure S4B). Effector molecules such as, e.g., IL-2, TNF-α and IFN-γ were upregulated in PBMCs but not T-cell-depleted PBMCs after restimulation. Analysis of the effects of dual immunosuppression (T+S/E/M/P) on the secretion of effector molecules (Supplementary Figure S5) revealed significantly reduced secretion of different effector molecules by PBMCs after stimulation with CMV_pp65 in presence of T+P (Supplementary Figure S5B). Overall, similar patterns were observed after stimulation under the influence of immunosuppression for EBV- and SARS-CoV-2-specific T cells (Supplementary Figure S5B).

Taken together, PRE and triple combinations significantly reduced activation and effector molecule secretion of CMV-specific T cells. While all CD4^+^ memory T-cell subsets were affected by triple combinations, effects on CD8^+^ T cells were mainly attributed to TEM. Among the double combinations, T+P had the most pronounced impact on antiviral T cells. Moreover, immunosuppressive treatment resulted in impaired T-cell responses towards EBV and SARS-CoV-2.

### TAC, MPA, PRE and Triple Combinations Inhibit Cytokine Production by CD4^+^ and CD8^+^ T-cell Subsets Upon Antigenic Stimulation

**FIGURE 2:**
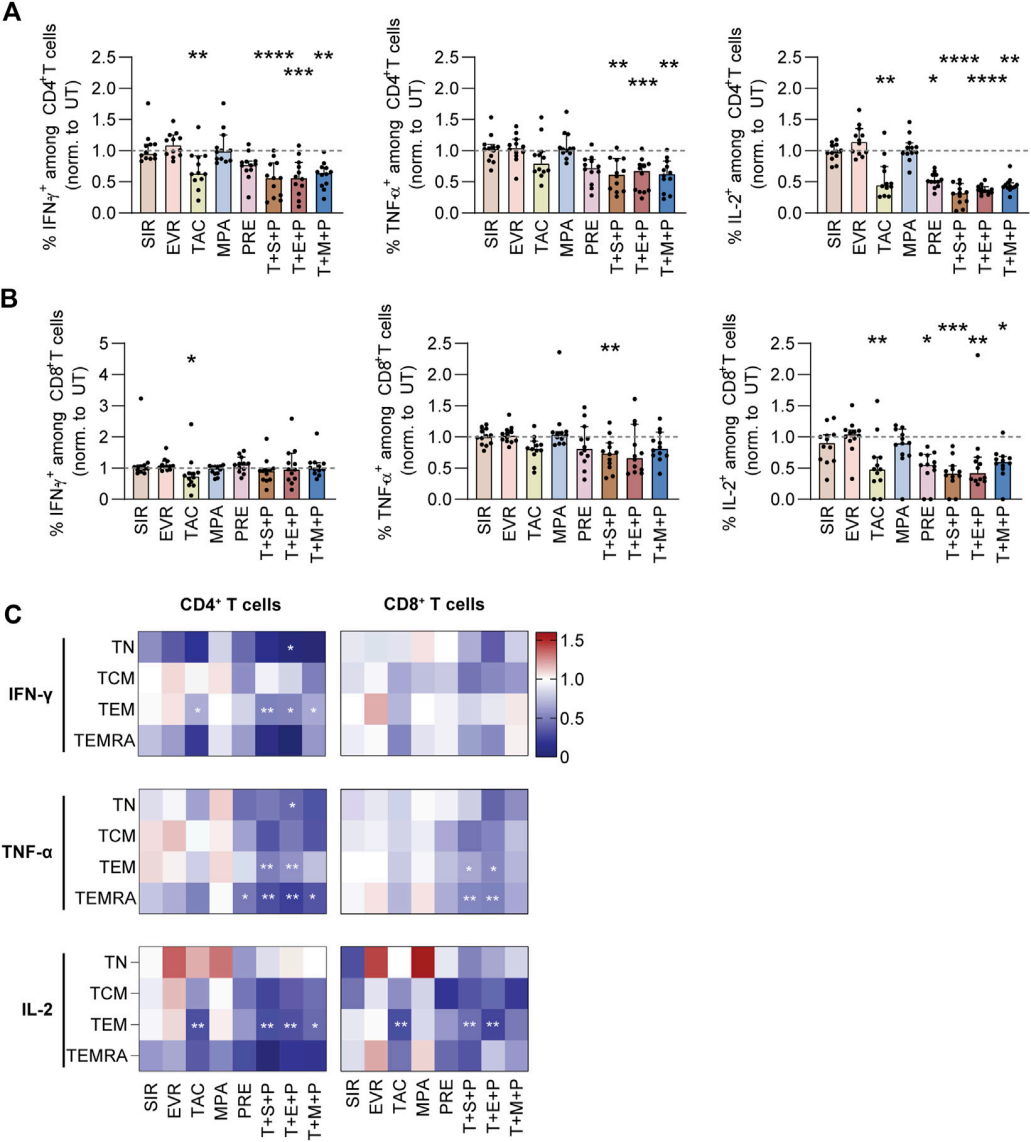
Cytokine profiling of CMV_pp65 stimulated PBMCs under immunosuppression. PBMCs were isolated from CMV+ donors, rested overnight and stimulated with CMV_pp65 on day 1 in presence and absence of indicated immunosuppressants. After 24h, intracellular cytokine production was detected using multicolor flow cytometry and secreted cytotoxic mediators were measured using a flow cytometry-based multiplex assay (LEGENDplex). **(A,B)** Bar graphs summarize frequencies of IFN-γ^+^, TNF-α^+^ and IL-2^+^ cells among **(A)** CD4^+^ and **(B)** CD8^+^ T cells. The data are shown as median and interquartile range Q1-Q3 (n = 12). **(C)** Heat maps summarize frequencies of IFN-γ^+^, TNF-α^+^ and IL-2^+^ cells among CD4^+^ (left) and CD8^+^ (right) memory T-cell subsets, normalized to untreated control (UT). Data are shown as median (n = 12). Statistical significance (in comparison to UT) was calculated using Friedman test followed by Dunn’s multiple comparison. *p < 0.05, **p < 0.01, ***p < 0.001, ****p < 0.0001. NC negative control (unstimulated), UT untreated, SIR/S sirolimus, EVR/E everolimus, TAC/T tacrolimus, MPA/M mycophenolic acid, PRE/P prednisolone, TN naïve T cells (CD45RA^+^/CD62L^+^), TCM central memory T cells (CD45RA^−^/CD62L^+^), TEM effector memory T cell (CD45RA^−^/CD62L^−^), TEMRA effector memory T cell re-expressing CD45RA (CD45RA^+^/CD62L^−^).

To further discriminate between CD4^+^ and CD8^+^ T cells, we performed intracellular cytokine staining of PBMCs stimulated with CMV_pp65 in absence or presence of immunosuppressants and triple combinations thereof (Figure 2; Supplementary Figure S6). The data were normalized to values obtained from UT controls (Figure 2; Supplementary Figure S6B, S6C). Frequencies of IFN-γ^+^, TNF-α^+^, and IL-2^+^ cells within CD4^+^ T cells were significantly reduced by triple combinations (Figure 2A). Moreover, IFN-γ^+^ cells within CD4^+^ T cells were significantly reduced by TAC and the frequencies of IL-2^+^ cells within CD4^+^ T cells were significantly reduced by TAC and PRE. In contrast, frequencies of IFN-γ^+^ cells within CD8^+^ T cells were reduced by TAC, whereas triple combinations had no impact (Figure 2B). TNF-α production by CD8^+^ T cells was slightly reduced in presence of triple combinations, while IL-2 production was significantly reduced by TAC, PRE and triple combinations. Inhibitory effects on CD4^+^ T cells were primarily focused on TEM (IFN-γ, TNF-α, IL-2) and TEMRA (TNF-α) (Figure 2C). Moreover, significantly reduced IFN-γ and IL-2 production by CD4^+^ TEM was observed in presence of TAC. Among CD8^+^ memory T-cell subsets, reduction of TNF-α and IL-2 production was comparable to CD4^+^ T-cell subsets.

Taken together, SIR and EVR mostly preserved the release of pro-inflammatory cytokines by CMV-specific memory T cells, which is in contrast to TAC, PRE and triple combinations. Moreover, impairment of IFN-γ production by immunosuppressive treatment was mostly restricted to CD4^+^ T cells, while IL-2 production was strongly reduced in CD4^+^ and CD8^+^ T cells.

### MPA and Triple Combinations Inhibit CMV-specific T-cell Proliferation

**FIGURE 3:**
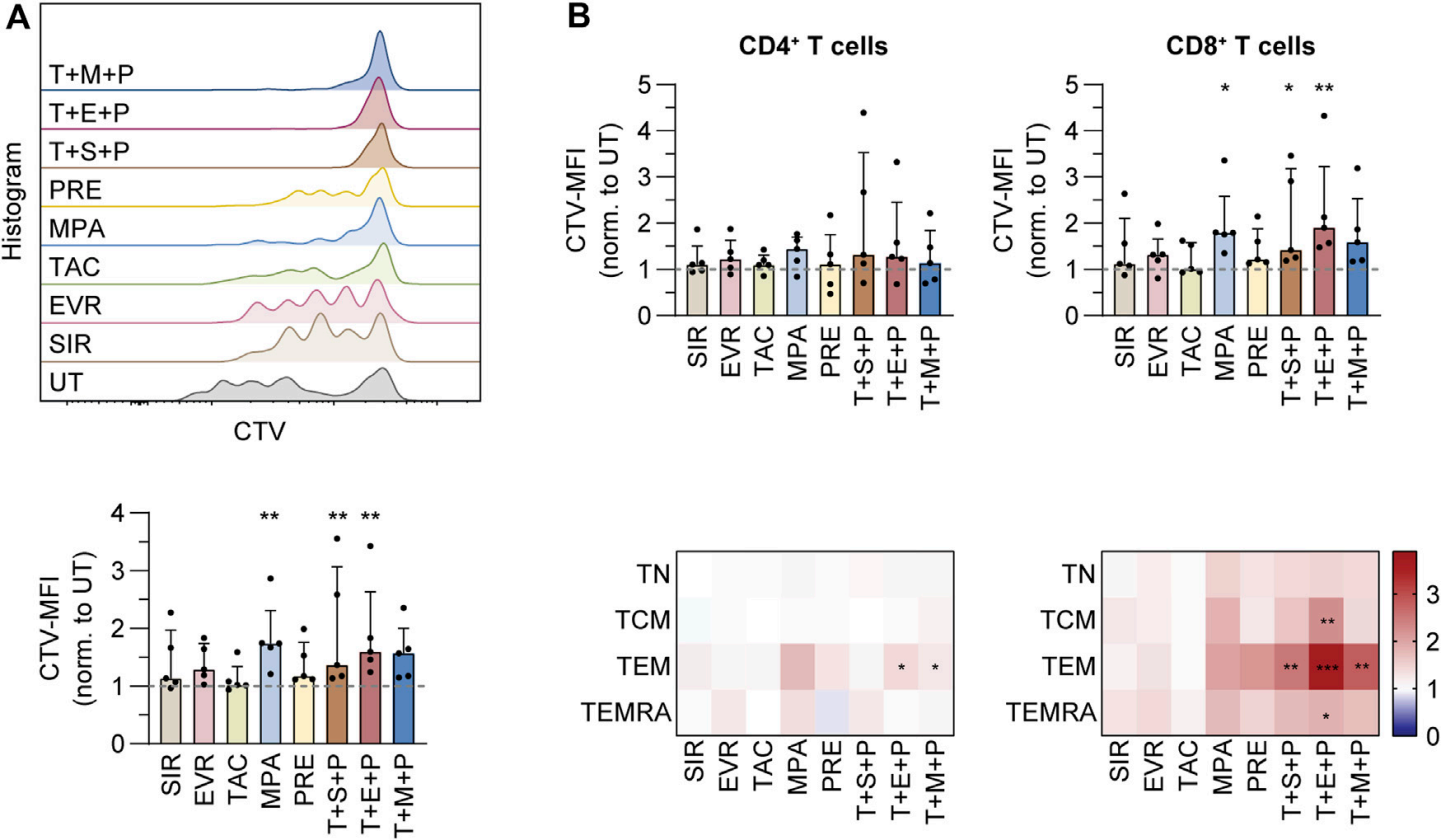
Proliferation analysis of purified CMV-specific T cells under immunosuppression. PBMCs were isolated from CMV+ donors, labeled with CellTrace™ Violet (CTV) and rested overnight, followed by magnetic enrichment of CMV-specific T cells using Cytokine Secretion Assay and CMV_pp65 stimulation. Afterwards, the T cells were expanded on irradiated autologous PBMCs in presence or absence of indicated immunosuppressants, followed by flow cytometric analysis. **(A)** Histograms showing CTV signals of CMV-specific T cells from a representative donor after 5 days of expansion (upper graph). Bar graph shows summarized mean fluorescent intensities (MFIs) of CTV from proliferating T cells on day 5, normalized to untreated control (UT) (lower graph). **(B)** Bar graphs show summarized MFIs of CTV from proliferating CD4^+^ (left) and CD8^+^ (right) T cells on day 5, normalized to untreated control (UT) (upper). Heat maps show summarized MFIs of CTV from proliferating CD4^+^ (left) and CD8^+^ (right) memory T-cell subsets on day 5, normalized to untreated control (UT) (lower). **(A,B)** Bar graphs show median and interquartile range Q1-Q3, each symbol represents data from one donor (n = 5). Heat maps show data as median values (n = 5). Statistical significance (in comparison to UT) was calculated for each T-cell subset using Friedman test followed by Dunn’s multiple comparison. *p < 0.05, **p < 0.01, ***p < 0.001. UT untreated, SIR/S sirolimus, EVR/E everolimus, TAC/T tacrolimus, MPA/M mycophenolic acid, PRE/P prednisolone, TN naïve T cells (CD45RA^+^/CD62L^+^), TCM central memory T cells (CD45RA^−^/CD62L^+^), TEM effector memory T cell (CD45RA^−^/CD62L^−^), TEMRA effector memory T cell re-expressing CD45RA (CD45RA^+^/CD62L^−^).

To analyze the impact of immunosuppression on proliferation of CMV-specific memory T cells, we isolated CMV_pp65-specific T cells by IFN-γ cytokine secretion assay (CSA). The cells were labeled with CellTrace Violet (CTV) proliferation dye and expanded on irradiated autologous PBMCs (feeder cells) in presence or absence of immunosuppressants and combinations thereof for 4 days (Figure 3; Supplementary Figure S7). The data were normalized to values obtained from untreated controls (Figure 3; Supplementary Figures S7B, S7C). Presence of MPA, T+S+P and T+E+P resulted in significantly reduced proliferation of T cells (Figure 3A). Among CD4^+^ T-cell subsets, treatment with T+E+P and T+M+P resulted in significantly reduced proliferation of TEM (Figure 3B). Proliferation of CD8^+^ TEM was significantly reduced in presence of all triple combinations and CD8^+^ TCM and TEMRA proliferation was significantly reduced in presence of T+E+P.

Taken together, treatment with MPA and triple combinations resulted in significantly impaired proliferation of CMV-specific T cells.

### PRE and Triple Combinations Impair CMV-specific T-cell Activation and Cytotoxicity

**FIGURE 4:**
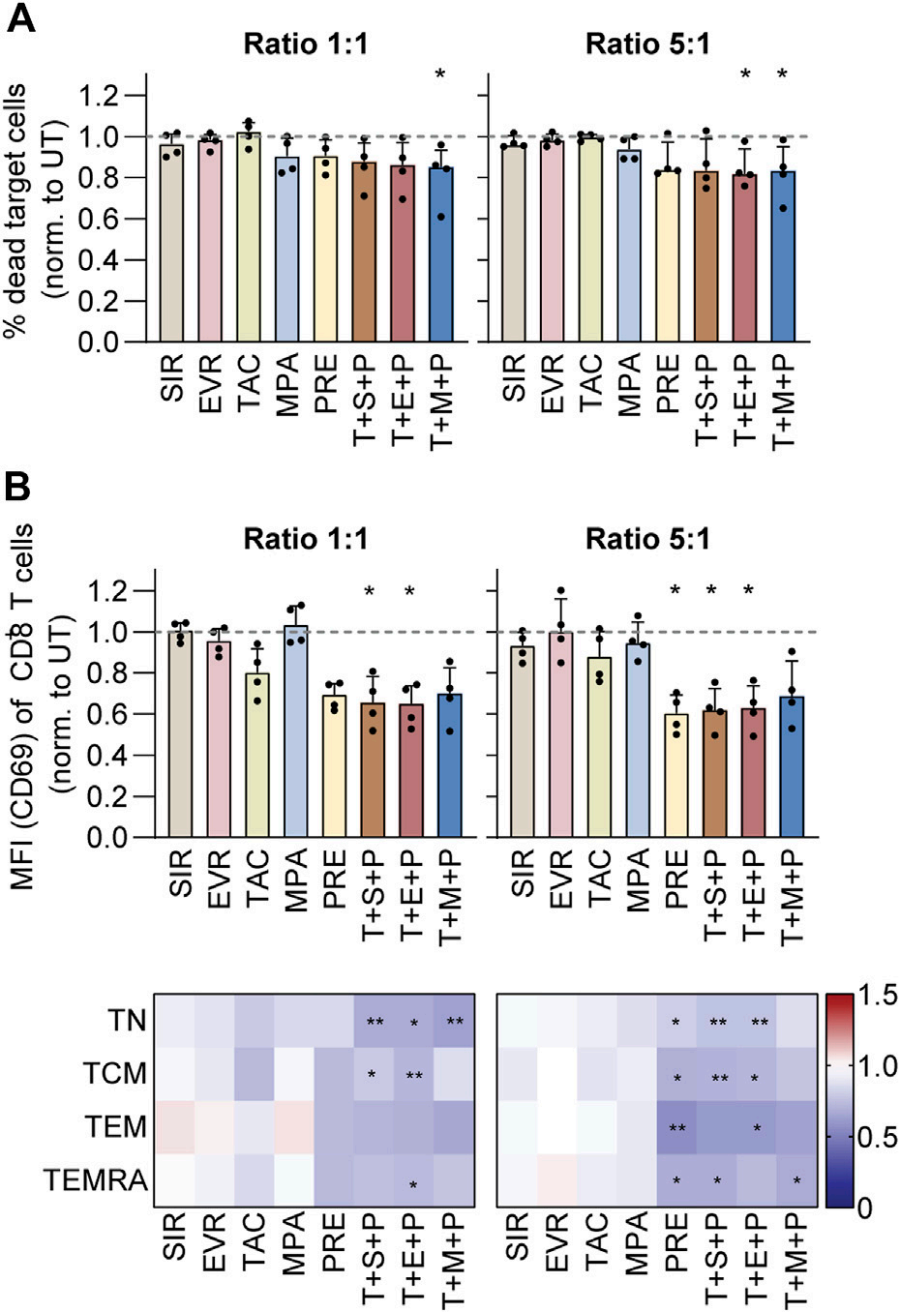
Cytotoxic capacity and activation of CMV-specific T cells under immunosuppression. PBMCs were isolated from CMV+ donors and rested overnight, followed by magnetic enrichment of CMV-specific T cells using Cytokine Secretion Assay and CMV_pp65 stimulation. The T cells were expanded on irradiated autologous PBMCs for 11 days and subsequently co-cultured with CTV-labeled autologous CMV_pp65-loaded PBMCs in different effector-to-target ratios and in presence or absence of indicated immunosuppressants. After 4 h their cytotoxic capacity was analyzed using flow cytometry. Unloaded PBMCs served as negative control. **(A)** Bar graphs show the frequencies of dead (7-AAD^+^) target cells, normalized to untreated control (UT). **(B)** Bar graphs show the CD69 expression (MFI) among CD8^+^ T cells, normalized to untreated control (UT) (upper). Heat maps show the CD69 expression (MFI) among CD8^+^ memory T-cell subsets, normalized to untreated control (UT) (lower). **(A,B)** Bar graphs show median and interquartile range Q1-Q3, each symbol represents data from one donor (n = 4). Heat maps show data as median values (n = 5). Statistical significance (in comparison to UT) was calculated using Friedman test followed by Dunn’s multiple comparison. *p < 0.05, **p < 0.01, ***p < 0.001. UT untreated, SIR/S sirolimus, EVR/E everolimus, TAC/T tacrolimus, MPA/M mycophenolic acid, PRE/P prednisolone, TN naïve T cells (CD45RA^+^/CD62L^+^), TCM central memory T cells (CD45RA^−^/CD62L^+^), TEM effector memory T cell (CD45RA^−^/CD62L^−^), TEMRA effector memory T cell re-expressing CD45RA (CD45RA^+^/CD62L^−^).

For measurement of the cytotoxic capacity of CMV-specific T cells under immunosuppression, CMV_pp65-specific memory T cells were isolated as described before and expanded on feeder cells for 12 days, followed by co-culture with CTV-labeled autologous CMV_pp65-loaded PBMCs in presence or absence of immunosuppressants. Unloaded PBMCs served as negative control. After 4 h, the cells were harvested for flow cytometric analysis of target cell death and T-cell activation (Figure 4; Supplementary Figure S8). The data were normalized to values obtained from UT controls (Figure 4; Supplementary Figure S8). While no unspecific cytotoxicity of T cells co-cultured with unloaded PBMCs was observed (Supplementary Figures S8A, S8B), frequencies of dead (7-AAD^+^) PBMCs were increased when peptide pool-loaded and co-cultured with T cells, and this effect was dose-dependent (Figure 4A; Supplementary Figure S8B). At both ratios, T+M+P resulted in reduced cytotoxicity of T cells towards loaded PBMCs. Moreover, at the 5:1 ratio, treatment with T+E+P significantly reduced cytotoxicity. Slightly reduced cytotoxicity was observed in presence of MPA, PRE and triple combinations at both ratios. In line, frequencies of CD69-expressing CD8^+^ T cells and memory subsets were significantly reduced under treatment with PRE (5:1), T+S+P (1:1 and 5:1) and T+E+P (1:1 and 5:1) (Figure 4B; Supplementary Figures S8C, S8D).

Taken together, PRE and triple combinations resulted in comparable inhibition of cytotoxicity and activation after co-culture with autologous CMV_pp65-loaded PBMCs.

### PRE and Triple Combinations Inhibit Real-Time Cytotoxicity Towards CMV-Infected Fibroblasts

**FIGURE 5:**
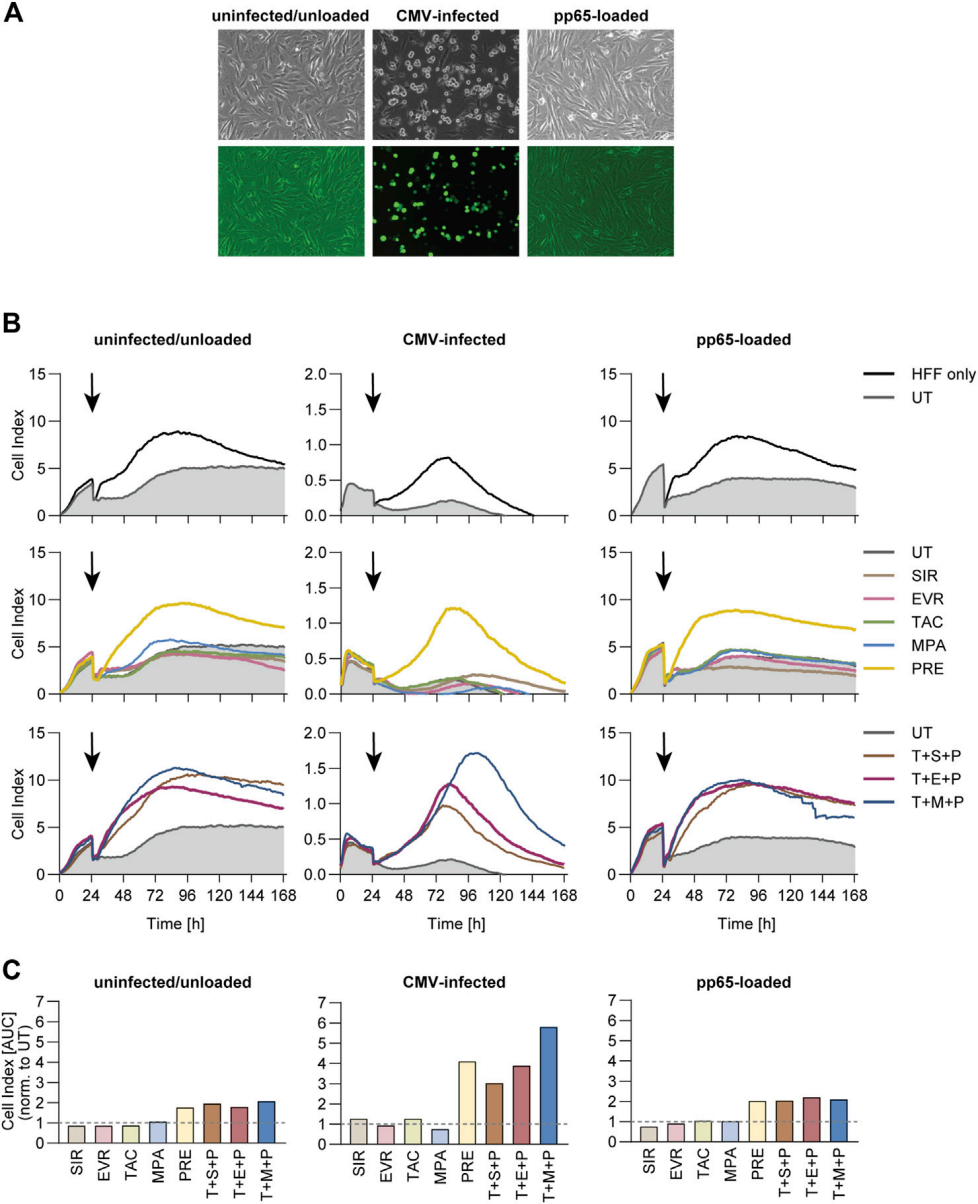
Cytotoxic capacity of CMV-specific T cells towards CMV-infected fibroblasts under immunosuppression. PBMCs were isolated from CMV+ donors and rested overnight, followed by magnetic enrichment of CMV-specific T cells using Cytokine Secretion Assay and CMV_pp65 stimulation. The T cells were expanded on irradiated autologous PBMCs for 11 days and subsequently co-cultured with uninfected, CMV-infected or CMV_pp65-loaded Human Foreskin Fibroblasts (HFF) in an effector-to-target ratio of 1:1 and in presence or absence of indicated immunosuppressants for 7 days using an xCELLigence RTCA S16 Real Time Cell Analyzer. **(A)** Microscopic image of the different target cells prior to co-culture. **(B)** Realtime impedance-based growth curves of HFF cells cultured alone (HFF cells only) or together with CMV-specific T cells in presence or absence of indicated immunosuppressants. Black arrows indicate time of T-cell addition. **(C)** Bar graphs display the AUC of growth curves shown in **(B)**, normalized to untreated control (UT). UT untreated, SIR/S sirolimus, EVR/E everolimus, TAC/T tacrolimus, MPA/M mycophenolic acid, PRE/P prednisolone.

To evaluate long-term effects of immunosuppressive treatment, we measured real-time cytotoxicity of CMV-specific T cells towards partially HLA-matched CMV-infected or CMV_pp65-loaded human foreskin fibroblasts (HFF) using xCelligence Real Time Cell Analyzer (RTCA) (Figure 5). Fluorescence microscopy confirmed the successful infection, indicated by expression of a green fluorescent protein (GFP) signal in the CMV-infected cells (Figure 5A). Direct comparison of growth curves for HFF cells only and HFF cells plus T cells showed reduced cell indices in presence of T cells for all three target cell conditions (Figure 5B). PRE and all triple combinations markedly inhibited cytotoxicity as indicated by higher cell indices. Area under the curve (AUC) values (Supplementary Figure S9A) were normalized to the AUC values obtained from the respective UT control (Figure 5C). While slightly higher normalized AUC values were measured in co-cultures treated with PRE or triple combinations, these effects were markedly stronger in co-cultures with CMV-infected HFF cells compared to the other two conditions.

Supernatants of these co-cultures were analyzed with respect to secreted cytotoxic mediators (Supplementary Figure S9B). Specific upregulation of IL-6, sFasL and IFN-γ was observed in co-cultures with CMV-infected HFF cells and this was slightly reduced in presence of PRE and triple combinations.

Taken together, CMV-specific T cells were unable to eliminate CMV-infected fibroblasts under immunosuppression with PRE or triple combinations, and this was accompanied by decreased effector molecule production.

### Summary

**FIGURE 6:**
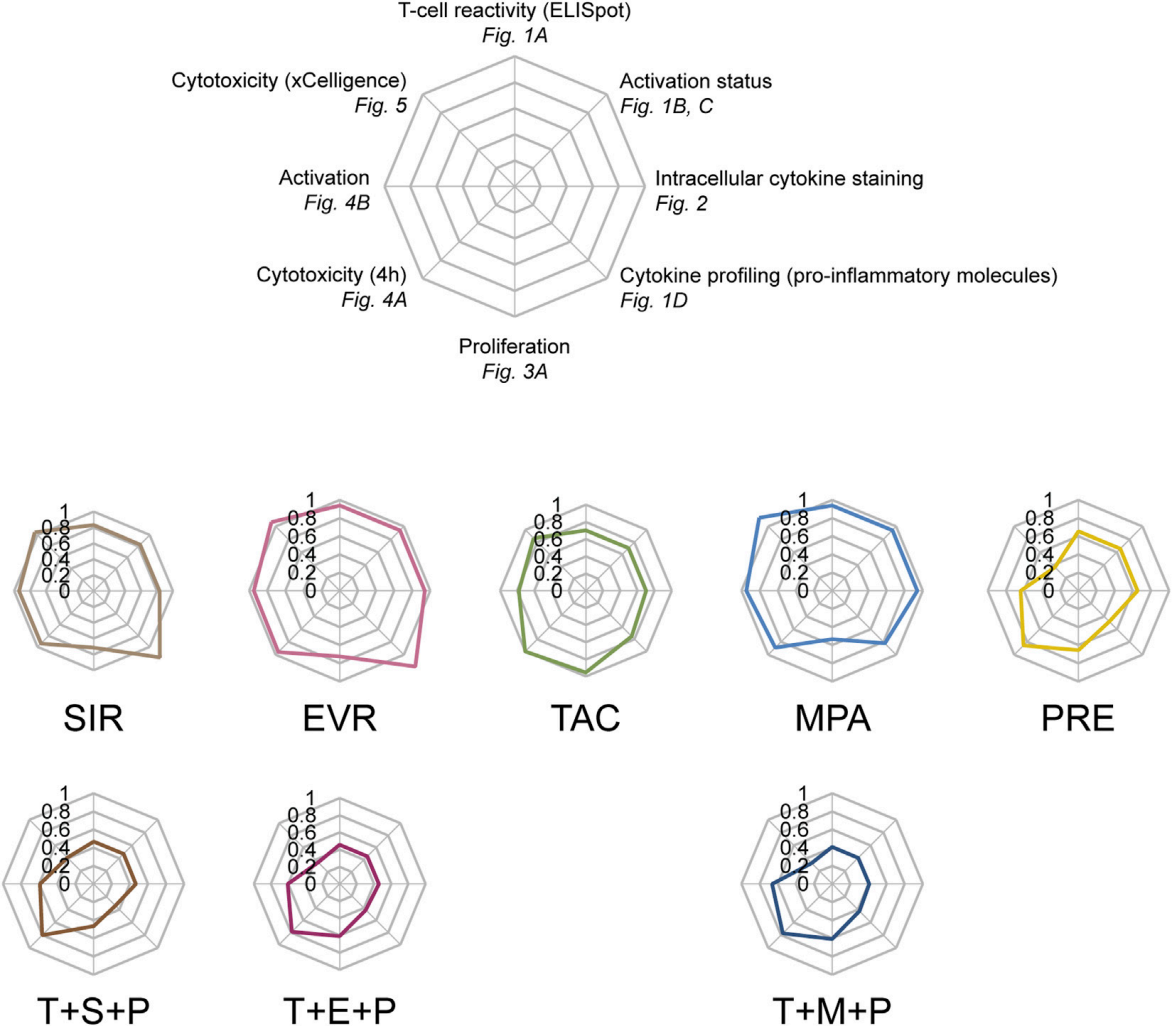
*In vitro* profiles of commonly used post-transplant immunosuppressants in context of antiviral T-cell immunity. Spider web graphs summarizing the impact of the respective immunosuppressants on CMV-specific T cells as measured by the indicated assays and in comparison to untreated controls. Values used for the diagrams are (clockwise starting from the top): IFN-γ ELISpot (spot numbers), activation status (frequencies of CD69^+^ CD4^+^ and CD8^+^ T cells), intracellular cytokine staining (cumulative frequencies of IFN-γ^+^/TNF-α^+^/IL-2^+^ CD4^+^ and CD8^+^ T cells), multiplex cytokine profiling (concentrations of pro-inflammatory molecules), proliferation (CD3^+^ T cells), cytotoxicity (4 h) (frequencies of dead target cells), activation (4 h) (CD69-MFIs of CD8^+^ T cells), realtime cytotoxicity (area under curve). UT untreated, SIR/S sirolimus, EVR/E everolimus, TAC/T tacrolimus, MPA/M mycophenolic acid, PRE/P prednisolone.

Spider web graphs including all assay read-outs were created for each immunosuppressant in comparison to UT controls (Figure 6). While all triple combinations conferred homogenously and broadly attenuated CMV-specific memory T cells, divergent effects of single immunosuppressants were observed. SIR and EVR slightly inhibited T-cell proliferation while mostly sparing activation and cytokine secretion. MPA selectively inhibited T-cell proliferation more profoundly. In contrast, TAC slightly inhibited different aspects of CMV-specific T-cell functionality and PRE had broad inhibitory effects on CMV-specific T cells.

## Discussion

The influence of post-transplant immunosuppressants on CMV susceptibility and on antiviral T cells is of high importance for choosing preventive and therapeutic measures, since T cells are required for the final control of CMV replication [31]. Appropriate T-cell function relies on different aspects such as proliferation, cytokine secretion and cytotoxicity [32] and these aspects are targeted via different mechanisms by post-transplant immunosuppressants. Usually, for early prevention of allograft rejection and perioperative lowering of maintenance immunosuppressants following SOT, an induction therapy is applied. In this phase, different T cell-depleting agents are used. However, most CMV diseases following SOT typically occur after 30–90 days [19–22]. At this point, mostly a switch to maintenance therapy has been made by using triple combinations [33, 34]. Of note, immunosuppressive regimens differ regarding choice of immunosuppressants and dosages between the transplanted organs and centers. Of note, in case of resistant/refractory CMV disease, treatment options include secondary antiviral drugs and individual change of immunosuppression [35]. In case of insufficient antiviral T-cell immunity, adoptive transfer of virus-specific T cells can restore a long-lasting endogenous antiviral immune defense [36, 37]. In this study, we screened commonly used immunosuppressive drugs and combinations thereof with respect to different aspects of T-cell functionality *in vitro*.

We observed that PRE and combinations containing PRE attenuate IFN-γ secretion, which is in harmony with earlier findings [38]. PRE, the active metabolite of prednisone, is a glucocorticoid with broad immunomodulatory effects including interference with different pro-inflammatory genes and non-genomic cytosolic molecule interferences [39, 40]. IFN-γ is crucially involved in the defense against CMV and it may foreshadow the outcome prior and post transplantation [41, 42] and determines the prognosis of critically ill patients as well [43]. It was recently demonstrated that addition of methylprednisolone to regimens featuring TAC and MMF worsened the T-cell response in liver transplant recipients [44]. We did not observe significant decreases of IFN-γ secretion by the other tested immunosuppressive drugs, which is in concordance especially for SIR and EVR [45]. Of note, an additive effect was revealed for triple combinations, exceeding the inhibitory potential of PRE. Additionally, PRE and triple combinations led to decreased expression of CD69, which is regulating T-cell differentiation and metabolism [46].

SIR and EVR are mTORi and interfere with a variety of cascades, including pathways essential for T-cell proliferation [47–50]. Despite their chemical difference, distinct pharmacokinetic characteristics and mTOR complex affinities have been summarized, creating the interest of detailed side-by-side comparisons [51]. Interestingly, clinical studies showed that mTORi-based regimens are associated with lower CMV infection incidences compared to MMF-based combinations [23–29, 52].

We extended the range of surveyed molecules using intracellular cytokine staining to measure IL-2 and TNF-α production, which are both known to play an important role in the anti-viral response [53, 54]. For the CNI TAC, which leads to a decreased activation of the nuclear factor of activated T cells (NFAT) and a lower production of pro-inflammatory stimuli [55–57], one of its main effects - the depletion of IL-2 - was reflected in our study. Furthermore, we found an inhibition pattern of TAC, PRE and triple combinations that was focused on TEM and TEMRA, which are known for secreting high amounts of cytokines [58].

Together with the production of pro-inflammatory molecules, recruitment and proliferation is required for T-cell mediated organ rejection [59] and therefore targeted by immunosuppressants. Here, MPA, the active metabolite of MMF, stood out in our study. As a purine synthesis inhibitor targeting the inosine-5′-monophosphate dehydrogenase (IMDPH), it is relatively lymphocyte specific, due to the compromised *de novo* pathway of guanosine nucleotides (lymphocytes cannot use salvage pathway of purine synthesis) and a high affinity to their IMDPH isoform. This leads to inhibited human T- and B-cell proliferation [60]. MPA has a high growth-arresting profile [61], which we conferred to be as effective as from the investigated triple combinations. Other groups described that its function extends beyond the antimetabolite pathway inhibition [62, 63], which was partly supported by our experiments, where it showed accompanying decreased cytokine release. For this, PRE and triple combinations showed severe T-cell impairment. Moreover, under triple combinations, slightly decreased cytotoxic capacity was observed, alongside reduction of T-cell activation.

Notably, the mTORi SIR and EVR showed a selective and compared to MPA less profound inhibition of CMV-specific T-cell proliferation. Our group showed earlier that SIR can augment CMV-specific effector memory T cells while inhibiting naive T cells [64], supporting the assumption that it does not only have an isolated immunosuppressive effect. Deciphering more mechanisms is a current topic, e.g., it was recently found that for kidney transplants, mTORi prevented CMV infection via αβ and γδ T-cell preservation [65]. Moreover, CMV seems to utilize mTOR for its replication, e.g., in macrophages [66]. Furthermore, for adoptive T-cell therapy, advanced strategies are being developed to overcome limitations due to immunosuppression, like the utilization of gene knockouts for creating T cell drug resistance [67, 68]. This displays an interesting approach besides providing evidence for individual changes to more favorable drugs regimens.

To evaluate functional effects of CMV-specific T cells in context of CMV infection, we established a real-time cytotoxicity model using CMV-infected human fibroblasts in which pp65 protein expression was reported as early as 1 h and up to 24 h post infection [69]. Here, we observed that PRE and triple combinations inhibited T cell-mediated elimination of CMV-infected fibroblasts, confirming our previous results. In a study by Jackson et al., CD8^+^ T cells recognizing peptides derived from different CMV proteins (pp65, IE-1) were effective in an *in vitro* virus dissemination assay independent of their peptide specificity [70], therefore indicating that the assay developed here can be utilized to investigate T-cell responses against different viral antigens. Such assays are of broad interest, e.g., for the investigation of chimeric antigen receptor (CAR) T cells [71] and may be beneficial for future projects studying virus-specific T cells as well.

Therapeutic drug monitoring is routinely applied for CNI/mTORi and occasionally for MMF/MPA to prevent rejection and toxicities. Hence, drug concentrations investigated in this study were derived from known plasma levels to mimic a clinical situation [72–75]. Immunosuppressive protocols vary between different institutions and patients, desired ranges of combinatory sustaining therapies may lie between 5–8 ng/mL of TAC, 3–8 ng/mL EVR and 1–3.5 μg/mL MPA, for example, following liver transplantation, which was represented in our study. In a recent publication, 7.5–20 mg/d administered PRE led to a median peak plasma concentration of 0.271–0.921 μg/mL [76]. While the concentration of PRE investigated in our study was above those concentrations applied during maintenance therapy, it rather correlates to early post-transplant oral dosage. Titration studies should be conducted in the future to allow for further conclusions on dose-dependent effects. However, the results of our screening study may be useful for these further studies, including clinical trials. Further experiments comparing alloreactivity and antiviral responses side-by-side may be helpful as well. In addition, a more detailed investigation of drug interferences is of great interest, since both, TAC and SIR/EVR, bind to the FK506 binding protein at first and thus may inhibit each other [77]. Moreover, only recall responses of memory T cells but not the activation of naïve T cells was analyzed, hence future studies are needed to investigate the dose-dependent effects on memory and naïve T cells. In this study, we aimed at systematic analysis of the impact of different immunosuppressive drugs on different aspects of antiviral T-cell functionality. The impact of different immunosuppressive treatment regimens in patients with different transplantation history needs to be addressed in future studies. Especially for SOT recipients at high risk, studies on the impact of immunosuppressive drugs on the initiation of an anti-CMV immune response via activation of naïve T cells are of great interest.

To conclude, we showed that immunosuppressants administered after SOT or HSCT differentially affect CMV-specific T-cell functionality. CMV-specific T-cell responses were strongly impaired by triple combinations, while SIR, EVR and MPA selectively affected T-cell proliferation. TAC slightly inhibited activation and cytokine production. Further, PRE strongly impaired CMV-specific memory T cells, which was also reflected in the investigated triple combinations. While the focus of this study was on the impact of immunosuppressive treatment on CMV-specific T-cell immunity, our data suggest that T-cell responses towards other clinically relevant viruses such as EBV and SARS-CoV-2 might be similarly–and in case of SARS-CoV-2 even more profoundly–affected by post-transplant immunosuppressive treatment. Based on our results on double combinations (T+S/E/M/P), it can be assumed that the discontinuation of PRE in patients receiving combinatory regimens such as T+S/E/M+P would be beneficial to restore antiviral T-cell immunity. Taken together, our data suggest potential beneficial effects of treatment with mTORi whilst, if possible, TAC, MPA, PRE and triple combinations should be used cautiously for patients at high risk or suffering from CMV disease.

## Data Availability

The original contributions presented in the study are included in the article/Supplementary Material, further inquiries can be directed to the corresponding author.
