# Supplementary material for: In Vitro Profiling of Commonly Used Post-transplant Immunosuppressants Reveals Distinct Impact on Antiviral T-cell Immunity Towards CMV
Source: Transpl Int. 2024 Apr 9;37:12720. doi: 10.3389/ti.2024.12720 (PMC11035762; doi:10.3389/ti.2024.12720)
Supplement: Supplementary file 1 [file DataSheet1.docx]

***In vitro* profiling of commonly used post-transplant immunosuppressants reveals distinct impact on antiviral T-cell immunity towards CMV**

*Running title: antiviral T cells and immunosuppression*

Markus Benedikt Krueger^1^, Agnes Bonifacius^1,2^, Anna Christina Dragon^1^, Maria Michela Santamorena^1^, Björn Nashan^3^, Richard Taubert^4^, Ulrich Kalinke^5^, Britta Maecker-Kolhoff^2,6^, Rainer Blasczyk^1^, Britta Eiz-Vesper^1,2*^

^1^ Institute of Transfusion Medicine and Transplant Engineering, Hannover Medical School, Hannover, Germany

^2^ German Center for Infection Research (DZIF), Site Hannover-Braunschweig, Germany

^3^ Clinic for Hepatopancreaticobiliary Surgery and Transplantation, First Affiliated Hospital, University of Science and Technology of China, Hefei, China

^4^ Department of Gastroenterology, Hepatology, Infectious Diseases and Endocrinology, Hannover Medical School, Hannover, Germany

^5^ Institute for Experimental Infection Research, TWINCORE, Centre for Experimental and Clinical Infection Research, A Joint Venture Between the Helmholtz Centre for Infection Research and Hannover Medical School, Hannover, Germany

^6^ Department of Pediatric Hematology and Oncology, Hannover Medical School, Hannover, Germany

MBK and AB contributed equally.

**Corresponding author**

Britta Eiz-Vesper

Carl-Neuberg-Str. 1

30625 Hannover

eiz-vesper.britta@mh-hannover.de

**Capsule Sentence Summary**

Systematic *in vitro* profiling of antiviral T cells under post-transplant immunosuppressants reveals favourable effects of mTOR inhibitors and strong impairment by prednisolone and combinatory regimens. This highlights the need for individualized immunosuppressive therapy to restore antiviral immunity in immunocompromised patients.

**Graphical Abstract**

The graphical abstract was created with BioRender.com.

**Materials and Methods**

**Isolation of PBMCs**

Healthy CMV+ donors were recruited from the Institute of Transfusion Medicine and Transplant Engineering, Hannover Medical School (MHH), Hannover, Germany. All donors gave their informed consent following approval by the Ethics Committee of MHH (ethics votes 3639_2017, 9001_BO-K, 9255_BO_K_2020). Peripheral blood mononuclear cells (PBMCs) were isolated from residual blood samples from platelet and plasma apheresis disposables used for routine collection by density gradient centrifugation.

**Immunosuppressants**

Immunosuppressants (all from Sigma-Aldrich, St. Louis MO, USA) were dissolved in DMSO (EMD Millipore Corp., Burlington MA, USA). For each drug, we used concentrations mostly known by a row of consensus reports and a comparable project investigating natural killer (NK) cells ^1-6^: sirolimus (SIR/S), everolimus (EVR/E), tacrolimus (TAC/T): 5 ng/ml, mycophenolic acid (MPA/M; clinically used is MMF, pro-drug of MPA): 1 µg/ml, prednisolone (PRE/P): 0.5 µg/ml, double (T+S/E/M/P) and triple combinations (T+S/E/M+P): concentrations as in single treated samples. Stimulated but drug-untreated specimens (UT) served as controls.

**Multicolor flow cytometry analysis**

If not stated otherwise, cells were washed with DPBS (Lonza, Verviers, Belgium), stained with the respective fluorochrome-labeled antibodies, incubated for 20 minutes in the dark at room temperature, washed again with DPBS, and optionally stained with 7-Aminoactinomycin D (7-AAD, BD Biosciences, Heidelberg, Germany) for 10 minutes in the dark. All samples were acquired on a 10-color BD FACS Canto (BD Biosciences).

**IFN-γ ELISpot assay under immunosuppression**

PBMCs were isolated on day 0 and rested overnight in RPMI-1640 (Lonza) supplemented with 10% heat-inactivated human AB serum (C.C.pro, Oberdorla, Germany) at 37 °C and 5% CO_2_. On day 1, cells were washed and transferred to pre-coated anti-IFN-γ ΕLISpot plates (Lophius Biosciences, Regensburg, Germany) at numbers of 2.5x10^5^ per well. Immunosuppressants were added and samples were stimulated with 1 µg/ml per peptide of CMV-phosphoprotein-65 (pp65) peptide pool (PepTivator CMV pp65, Miltenyi Biotec, Bergisch Gladbach, Germany), Epstein-Barr Virus (EBV) Consensus peptide pool (PepTivator EBV Consensus, Miltenyi Biotec, Bergisch Gladbach) or SARS-CoV-2 Spike (S) peptide pool (PepMix SARS-CoV-2 (Spike B.1.1.529 / BA.1 / Omicron), JPT technologies, Berlin, Germany). Following overnight incubation, spots were developed using alkaline phosphatase-conjugated monoclonal anti-IFN-γ antibody (Mabtech, Sweden) and 5-Bromo-4-chloro-3-indolyl phosphate/nitro blue tetrazolium chloride (BCIP/NBT, Serva, Heidelberg, Germany) substrate. Spots were counted using an automated reader (AID iSpot spectrum, AID EliSpot 7.0 Software, AID, Strassberg, Germany) and average values were calculated from duplicate wells. Average spot intensities and spot sizes were analyzed using the AID EliSpot 7.0 Software (AID, Strassberg, Germany).

**Activation profile of antiviral T cells under immunosuppression**

PBMCs were isolated, rested overnight and seeded at 1x10^6^ per well in 96-well suspension plates (Sarstedt, Nümbrecht, Germany). Cells were stimulated on the following day with CMV_pp65, EBV_Consensus, or SARS-CoV-2_S peptide pool and cultured in presence and absence of immunosuppressants. Unstimulated cells served as negative controls. After 24 hours, supernatants were collected and stored at -20 °C until further processing. The cells were harvested and stained for flow cytometric analysis: 7-AAD (BD Biosciences), anti-CD3 FITC (7A2), anti-CD4 AlexaFluor700 (SK3), anti-CD8 APC (SK1), anti-CD45 APC/Cy7 (2D1), anti-CD45RA BV510 (HI100), anti-CD62L PE/Cy7 (DREG-56), anti-CD25 PE (BC96), anti-CD69 BV605 (FN50) (all BioLegend, San Diego, USA).

**T-cell depletion**

PBMCs were isolated from healthy CMV+ donors and rested overnight in TexMACS (Miltenyi Biotec) at 1x10^7^ cells/ml at 37 °C and 5% CO_2_. CD8^+^/CD4^+^ T-cell depletion was performed by magnetic cell sorting (MACS) using CD8 and CD4 Microbeads (Miltenyi Biotec) following the manufacturer’s instructions. T-cell depleted fractions (PBMC-depl) were collected using LD columns (Miltenyi Biotec) and rested overnight. PBMCs and PBMC-depl were analyzed using multicolor flow cytometry using the following antibodies: anti-CD45 APC/Cy7 (2D1), anti-CD3 FITC (SK7), anti-CD4 Alexa Fluor700 (SK3) and anti-CD8 APC (SK1) (all BioLegend). The following day cells were harvested, counted and seeded at the density of 1x10^6^ per well in 96-well plates. Cells were stimulated with CMV_pp65 peptide pool (Peptivator CMVpp65, Miltenyi Biotec) at a final concentration of 1 µg/ml. Unstimulated cells served as negative control. After 24 hours of incubation, supernatants were collected and stored at -20°C for subsequent multiplex analysis.

**Multiplex cytokine analysis of antiviral T cells under immunosuppression**

Supernatants were analyzed using LEGENDplex™ CD8/NK and HU Immune Checkpoint Panel 1 kits according to the manufacturer’s instructions (BioLegend). Samples were acquired on a 10-color BD FACS Canto (BD Biosciences).

**Cytokine production of CMV-specific memory T-cell subsets under immunosuppression**

PBMCs were isolated, rested overnight and seeded at 1x10^6^ per well in 96-well suspension plates (Sarstedt). Cells were stimulated on the following day with CMV_pp65 peptide pool and cultured in presence and absence of immunosuppressants. Unstimulated cells served as negative controls. After one hour of stimulation, Brefeldin A (BioLegend,) was added. Samples were collected on day 2 for fixation and staining using IntraPrep Permeabilizaton Reagent (Beckman Coulter, Brea, USA). Surface antibodies and Zombie NIR viability dye (BioLegend) were added according to the manufacturer's instructions. Intracellular antibodies were added after permeabilization and cells were analyzed via flow cytometry. Antibodies used were the following: anti-CD4 PerCP (SK3), anti-CD8 PE/Cy7 (SK1), anti-CD45 Pacific Blue (2D1), anti-CD45RA BV605 (HI100), anti-CD62L BV510 (DREG-56), anti-IFN-γ FITC (4S.B3), anti-TNF-α PE (Mab11), anti-IL-2 APC (MQ1-17H12) (all BioLegend).

**Isolation of CMV-specific T cells by Cytokine Secretion Assay**

The isolation of CMV-specific CD4^+^ and CD8^+^ T cells was performed as described before^7^. In brief, PBMCs were isolated from CMV+ healthy donors, rested overnight and restimulated with overlapping peptide pool CMV_pp65 (1 µg/ml per peptide; Miltenyi Biotec). After 4h, IFN-γ-producing cells were detected and magnetically enriched using the the IFN-γ Secretion Assay - Cell Enrichment and Detection Kit (Miltenyi Biotec) according to the manufacturer’s instruction. Purity of obtained CMV-specific T cells was confirmed using multicolor flow cytometry: 7-AAD (BD Biosciences), anti-IFN-γ PE (contained in CSA kit), anti-CD45 APC/C7 (sD1), anti-CD3 FITC (SK1), anti-CD4 AlexaFluor700 (SK3), anti-CD8 APC (SK7), anti-CD45RA BV605 (HI100), anti-CD62L PE/Cy7 (DREG-56) (all BioLegend).

**Proliferation analysis of CMV-specific T cells under immunosuppression**

PBMCs were isolated on day 0, a fraction of them was labeled with CellTrace Violet™ proliferation dye (CTV, Thermo Fisher Scientific, Waltham, USA) and rested overnight. On day 1, unlabeled cells were irradiated at 30 Gray (feeder cells), while CTV-labelled cells were used for the enrichment of CMV-specific T cells as described above. These cells were then co-cultured in a T/feeder-cell-ratio of 1:100, supplemented with immunosuppressants and cultured in TexMACS™ medium at 37 °C, 5% CO_2_. The cells were split 1:2 on day 3, refilled with medium and drugs and harvested on day 5 for microscopy counting and flow cytometry analysis: 7-AAD (BD Biosciences), anti-CD3 FITC (7A2), anti-CD4 AlexaFluor700 (SK3), anti-CD8 APC (SK1), anti-CD45 APC/Cy7 (2D1), anti-CD45RA BV605 (HI100), anti-CD62L PE/Cy7 (DREG-56) (all BioLegend).

**4h-cytotoxicity assay of CMV-specific T cells under immunosuppression**

Following PBMC isolation CMV-specific T cells were isolated the next day, co-cultured 1:100 with autologous irradiated PBMCs and supplemented with IL-7 and IL-15 (both Peprotech, Hamburg, Germany), each at 10 ng/ml. Another part of PBMCs was frozen at -80 °C. The T-cell culture was split and refilled when reaching high cell confluence or medium color change. On day 11, the PBMCs were thawed, labelled with CellTrace Violet™ (Thermo Fisher Scientific) and one part was loaded with CMV_pp65 peptide pool (Miltenyi Biotec) overnight. Expanded CMV-specific T cells were harvested, washed and incubated overnight in immunosuppressants. On day 12, the T cells and PBMCs were co-cultured at ratios of 1:1 and 5:1 with a total cell number of 2x10^5^ cells per well in 96-well plates (Sarstedt) in TexMACS™ supplemented with 3% AB serum and immunosuppressants. After 4 hours, cells were harvested and stained for flow cytometric analysis: 7-AAD (BD Biosciences), anti-CD3 FITC (7A2), anti-CD4 AlexaFluor700 (SK3), anti-CD8 APC (SK1), anti-CD45 APC/Cy7 (2D1), anti-CD45RA BV510 (HI100), anti-CD62L PE/Cy7 (DREG-56), anti-CD25 PE (BC96), anti-CD69 BV605 (FN50) (all BioLegend).

**Real-time cytotoxicity assay of CMV-specific T cells under immunosuppression**

Human Foreskin Fibroblasts (HFF cells; kindly provided by M. Messerle, Hannover Medical School) were cultured in DMEM plus optimized medium (FibroGRO, all Merck/Sigma-Aldrich, Darmstadt, Germany). Partially HLA-matched CMV-specific T cells were enriched and expanded as described above. On day 0, HFF cells were plated in 6-well plates (TPP Techno Plastic Products AG, Trasadingen, Switzerland) and infected with human cytomegalovirus RV-TB40-BACKL7-SE-UL40rep^8^ (kindly provided by K. Laib Sampaio and C. Sinzger, Ulm University Medical Center, Ulm, Germany) at a multiplicity of infection of 3 on day 1. Following that, they were seeded into E-Plates 16 PET and placed in an xCELLigence RTCA S16 Real Time Cell Analyzer (ACEA Biosciences, San Diego, CA, USA) for achieving adherence prior to T-cell contact. On day 2, CMV-specific T cells were added at a ratio of 1:1 and cultures were supplemented with immunosuppressants, measurement then went on for 6 days.

**Visualization and statistical analysis**

Data were analyzed using FlowJo_v10.7.1 (BD Biosciences), LEGENDplex V.8.0 software (BioLegend) and GraphPad Prism v8 (GraphPad Software Inc., San Diego, CA, USA). Results are displayed as medians with interquartile ranges from Q1 (25%) – Q3 (75%) if not stated otherwise. Statistical analysis is described in the respective figure legends.

**Supplemental Figures**


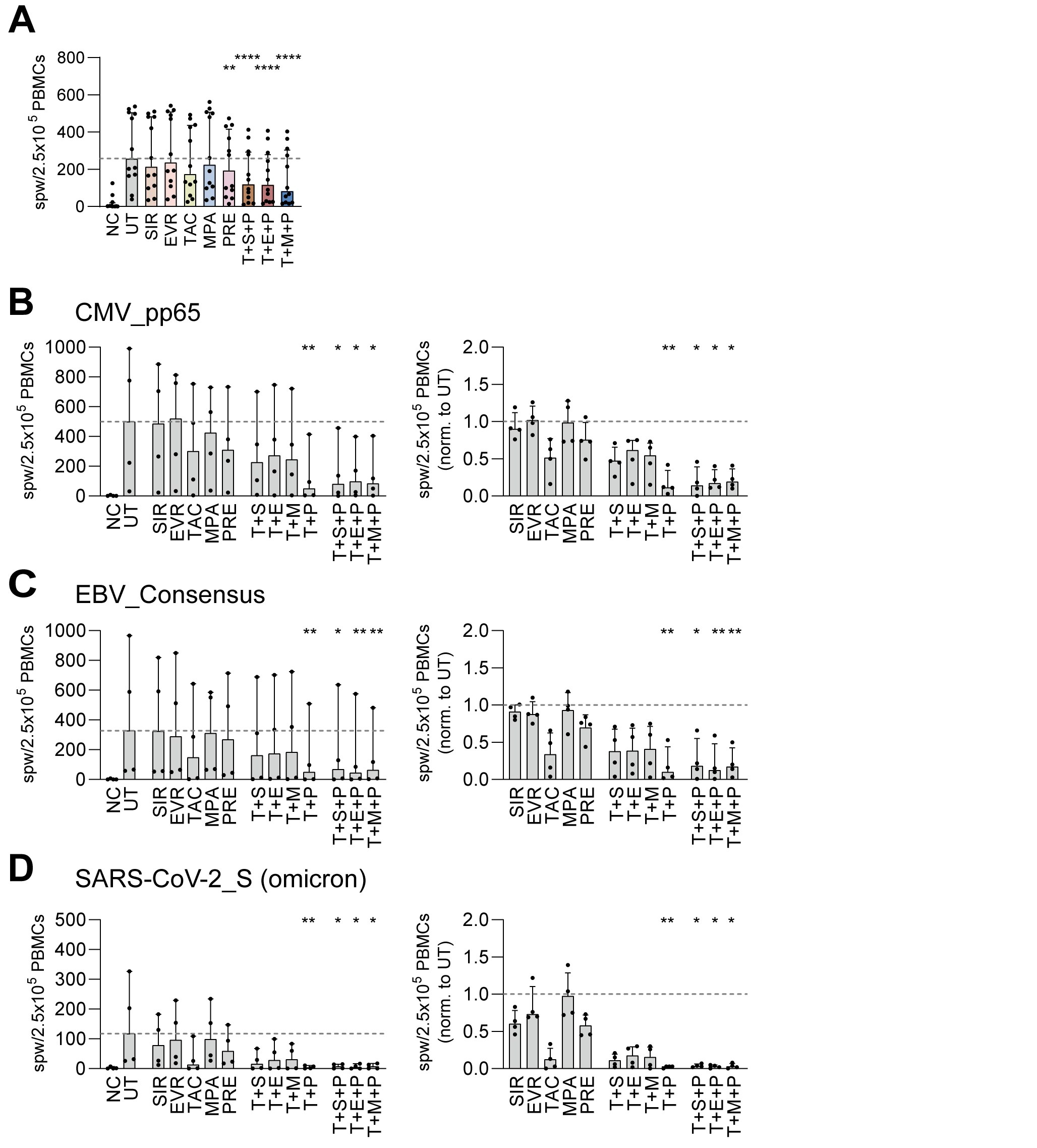


**Figure S1: Functionality of antiviral T cells under immunosuppression.** PBMCs were isolated from CMV+ donors, rested overnight and stimulated with indicated peptide pools on day 1 in presence and absence of indicated immunosuppressants on IFN-γ ELISpot plates. After 24h, secreted IFN‑γ was detected on ELISpot plates. Summarized IFN-γ ELISpot results are shown as spots per well (spw)/2.5x10^5^ PBMCs. Bar graphs show median and interquartile range Q1-Q3, each symbol represents data from one donor. **(A)** n=12, **(B.D)** n=4. Statistical significance (in comparison to UT) was calculated using Friedman test followed by Dunn’s multiple comparison. *p<0.05, **p<0.01, ***p<0.001. UT untreated, SIR/S sirolimus, EVR/E everolimus, TAC/T tacrolimus, MPA/M mycophenolic acid, PRE/P prednisolone.


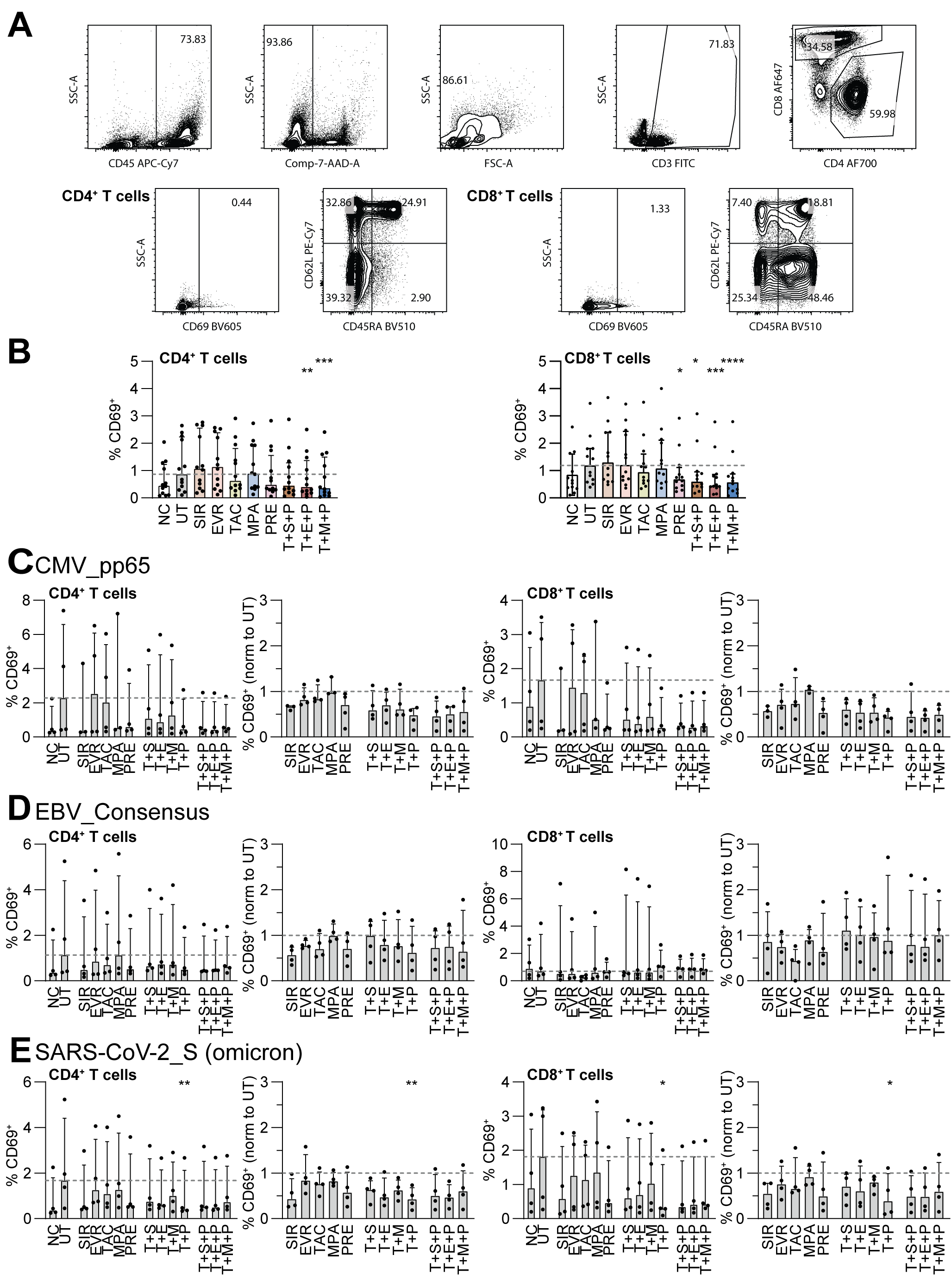


**Figure S2: Activation of antiviral T cells under immunosuppression.** PBMCs were isolated from CMV+ donors, rested overnight and stimulated with indicated peptide pools on day 1 in presence and absence of indicated immunosuppressants. After 24h, cells were harvested for flow cytometric analysis. **(A)** Gating strategy for analysis of CD69+ cells among CD4+ and CD8+ T cells. **(B-E)** Frequencies of CD69^+^ cells among CD4^+^ T cells and CD8^+^ T cells. Bar graphs show median and interquartile range Q1-Q3, each symbol represents data from one donor. **(B)** n=12, **(C-E)** n=4. Statistical significance (in comparison to UT) was calculated using Friedman test followed by Dunn’s multiple comparison. *p<0.05, **p<0.01, ***p<0.001, ****p<0.0001. UT untreated, SIR/S sirolimus, EVR/E everolimus, TAC/T tacrolimus, MPA/M mycophenolic acid, PRE/P prednisolone.


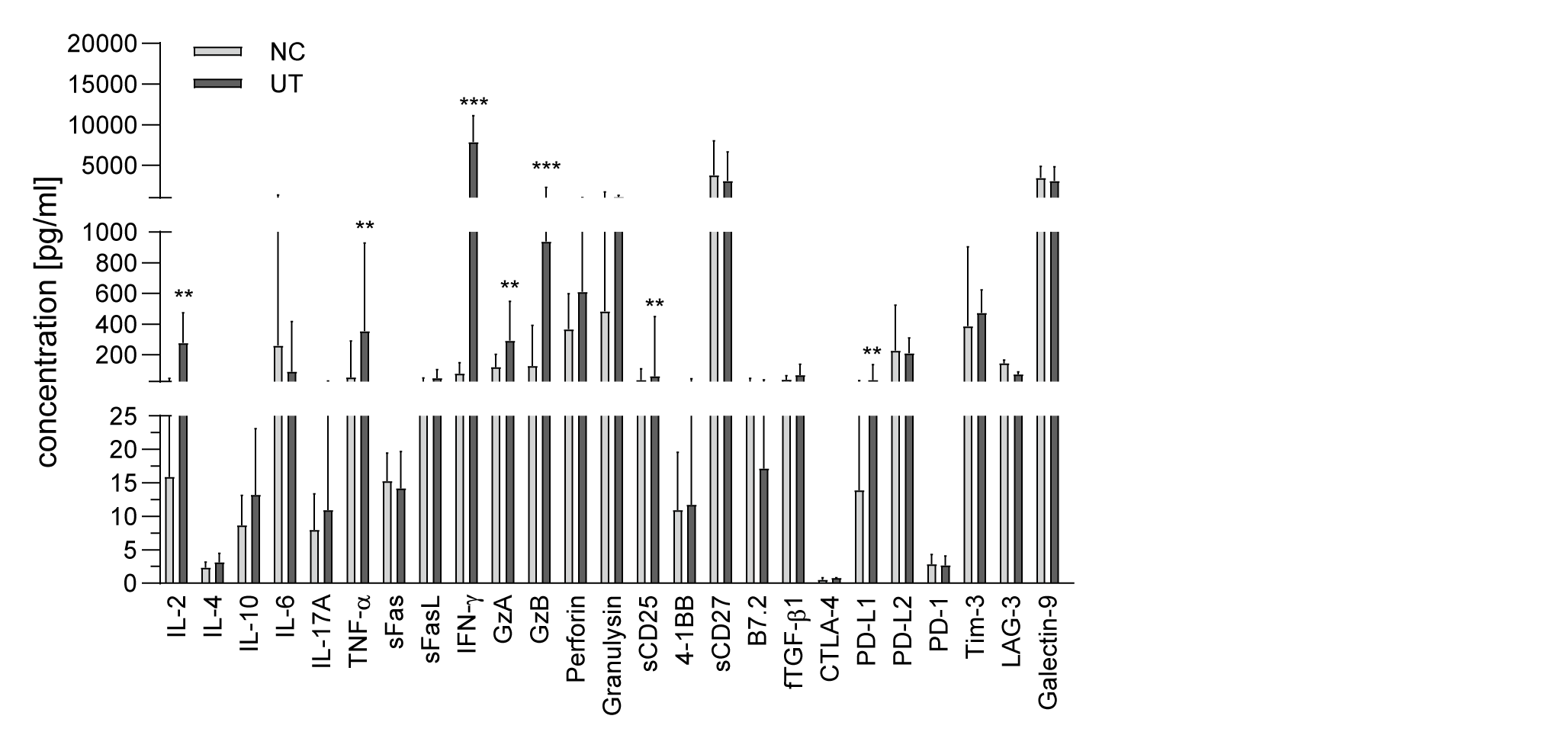


**Figure S3: Effector molecule secretion by antiviral T cells.** PBMCs were isolated from CMV+ donors, rested overnight and stimulated with CMV_pp65 on day 1. After 24h, cell culture supernatants were harvested for multiplex analysis. Bar graph shows the concentration of indicated analytes in culture supernatants from unstimulated (negative control, NC) and CMV_pp65-stimulated (untreated control, UT) PBMCs. Data are shown as median and interquartile range Q1-Q3. Statistical significance was calculated using Wilcoxon matched-pairs signed rank test (n=12). *p<0.05, **p<0.01, ***p<0.001. UT untreated, SIR/S sirolimus, EVR/E everolimus, TAC/T tacrolimus, MPA/M mycophenolic acid, PRE/P prednisolone.


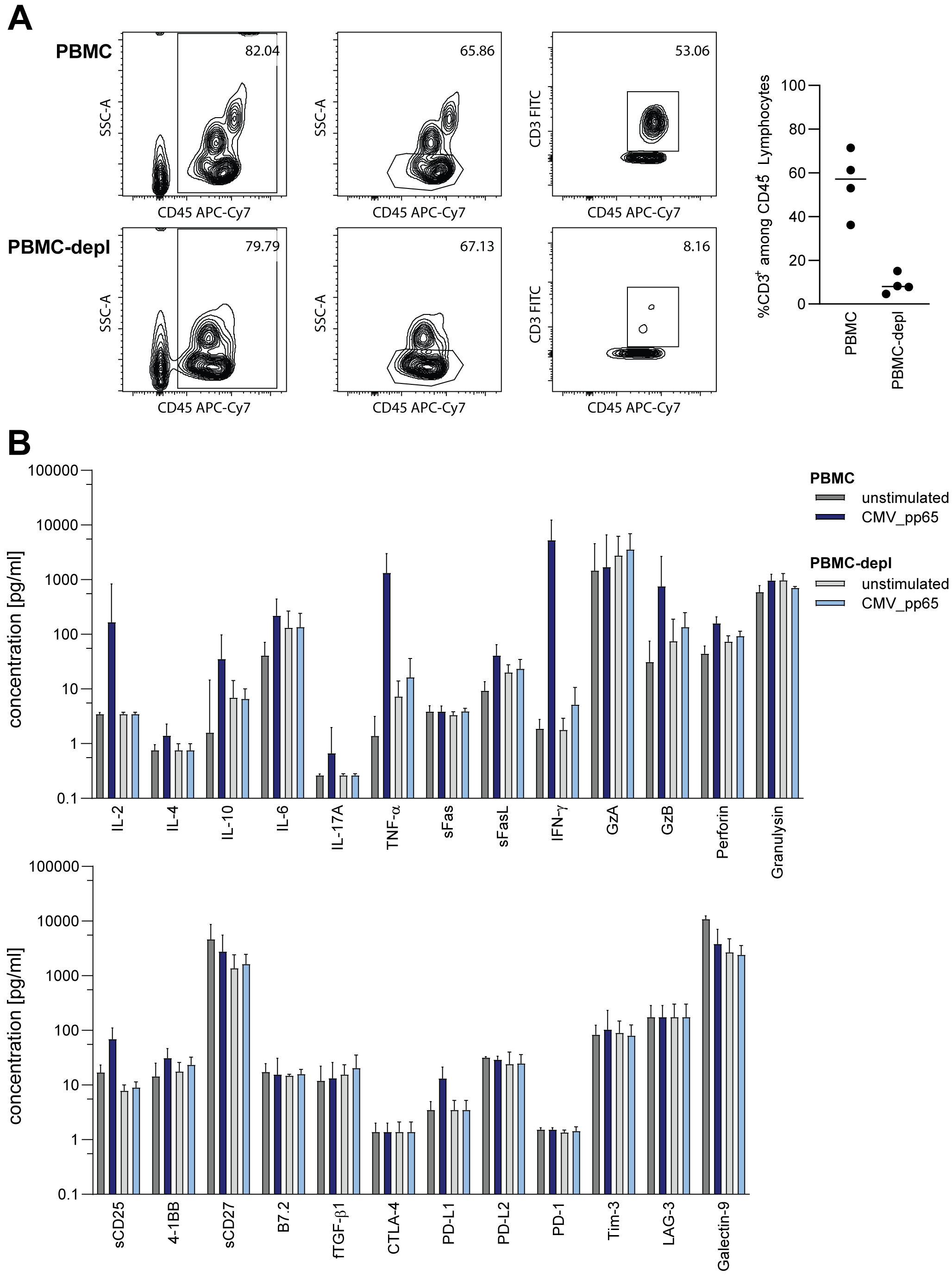


**Figure S4: T-cell depletion assay.** PBMCs were isolated from CMV+ donors (n=4) and T cells were depleted by MACS (PBMC-depl). Both cell fractions (PBMC and PBMC-depl) were rested overnight and stimulated with CMV_pp65 on day 1. After 24h, cell culture supernatants were harvested for multiplex analysis. **(A)** Flow cytometric analysis of PBMC and PBMC-depl. **(B)** Bar graph shows the concentration of indicated analytes in culture supernatants from unstimulated and CMV_pp65-stimulated (untreated control, UT) PBMCs and PBMC-depl (PBMC w/o T cells). Data are shown as median and interquartile range Q1-Q3.


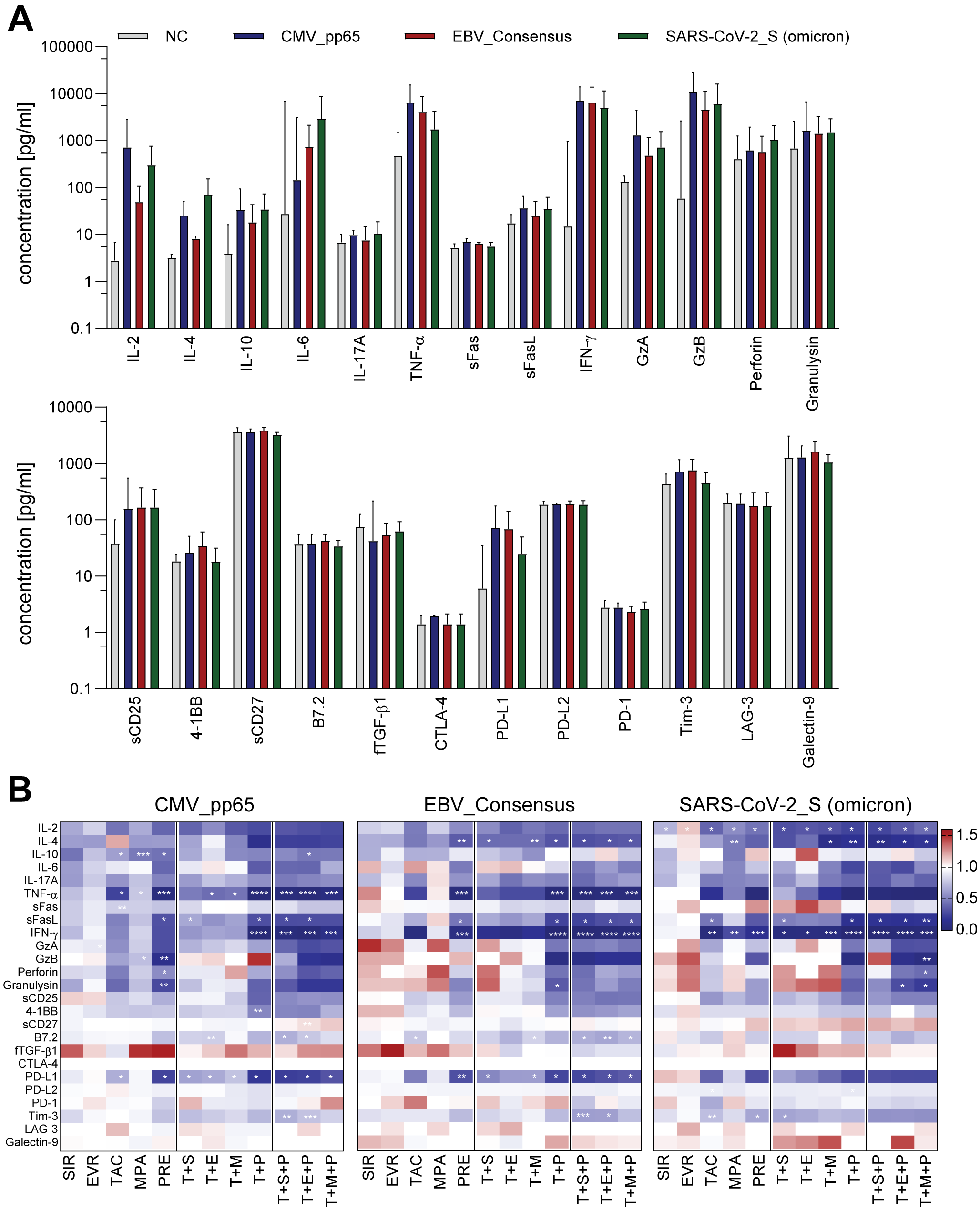


**Figure S5: Effector molecule secretion by antiviral T cells under immunosuppression.** PBMCs were isolated from CMV+ donors (n=4). Cells were rested overnight, followed by stimulation with indicated peptide pools on day 1 in presence or assence of indicated immunosuppressants. After 24h, cell culture supernatants were harvested for multiplex analysis. **(A)** Bar graph shows the concentration of indicated analytes in culture supernatants. Data are shown as median and interquartile range Q1-Q3. **(B)** Heat maps show median values, normalized to UT (n=4). Statistical significance (in comparison to UT) was calculated using 2way ANOVA followed by Dunnett’s multiple comparison. *p<0.05, **p<0.01, ***p<0.001, ****p<0.0001. NC negative control (unstimulated), UT untreated, SIR/S sirolimus, EVR/E everolimus, TAC/T tacrolimus, MPA/M mycophenolic acid, PRE/P prednisolone.


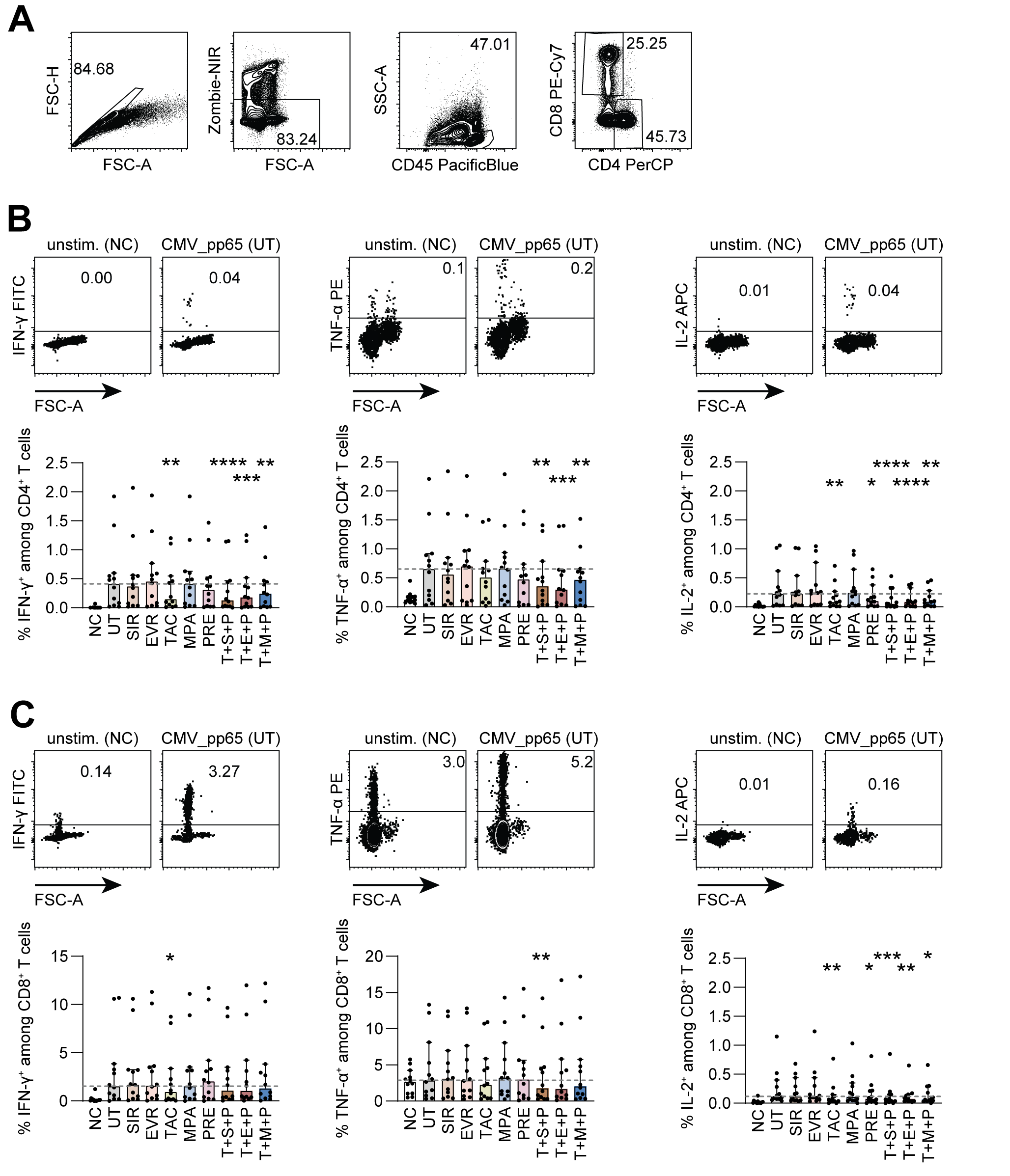


**Figure S6: Cytokine production of CMV-specific T cells under immunosuppression.** PBMCs were isolated from CMV+ donors, rested overnight and stimulated with CMV_pp65 on day 1 in presence and absence of indicated immunosuppressants. After 24h, intracellular cytokine production was detected using multicolor flow cytometry. **(A)** Gating strategy for analysis of IFN-γ^+^, TNF-α^+^ and IL-2^+^ cells among CD4^+^ and CD8^+^ T cells. **(B-C)** Exemplary FACS plots and summarizing graphs. Bar graphs show frequencies of IFN-γ^+^, TNF-α^+^ and IL-2^+^ cells among **(B)** CD4^+^ and **(C)** CD8^+^ T cells. The data are shown as median and interquartile range Q1-Q3 (n=12). Statistical significance (in comparison to UT) was calculated using Friedman test followed by Dunn’s multiple comparison. *p<0.05, **p<0.01, ***p<0.001, ****p<0.0001. NC negative control (unstimulated), UT untreated, SIR/S sirolimus, EVR/E everolimus, TAC/T tacrolimus, MPA/M mycophenolic acid, PRE/P prednisolone.


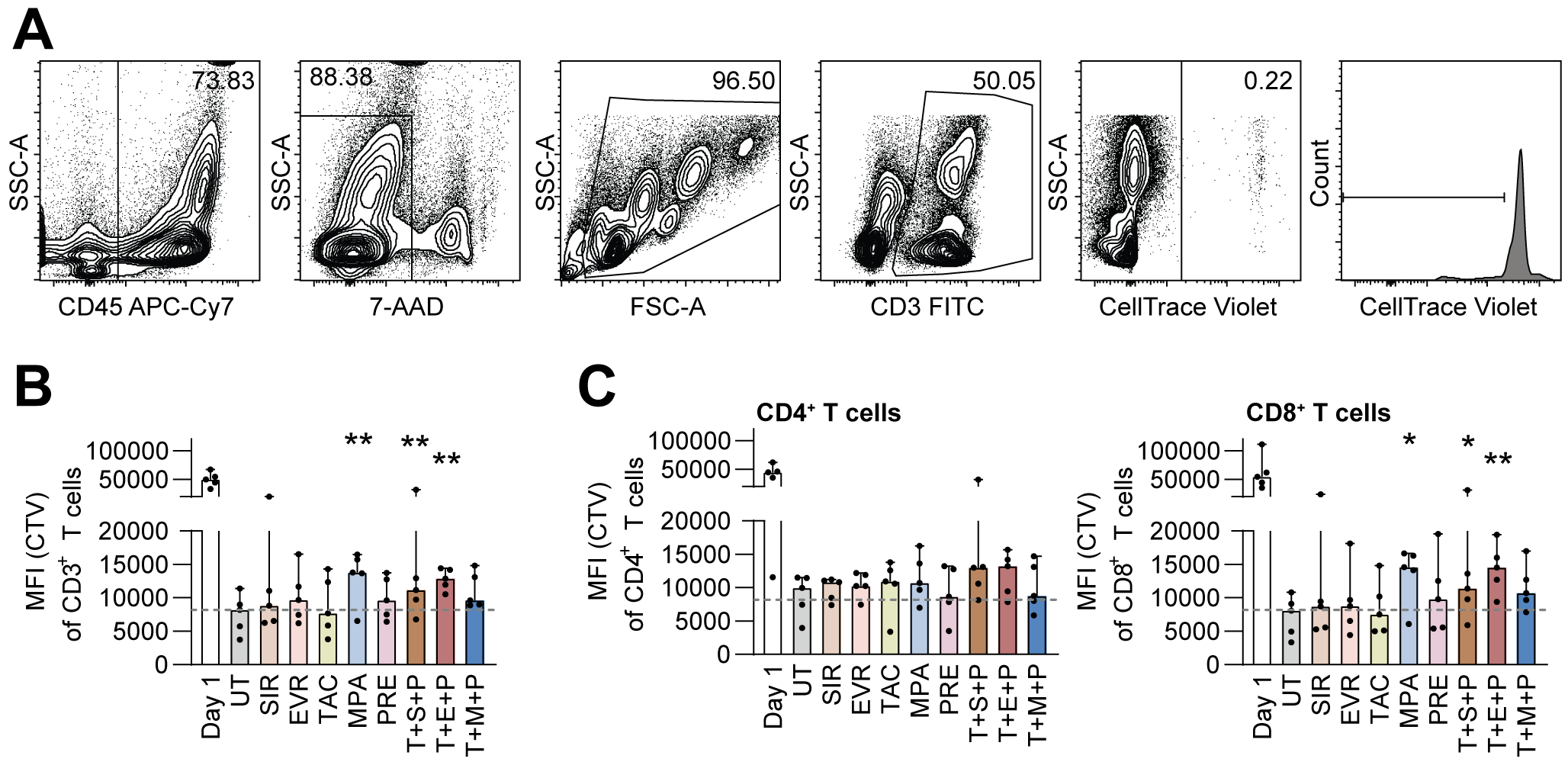


**Figure S7: Proliferative capacity of CMV-specific T cells under immunosuppression.** PBMCs were isolated from CMV+ donors, labeled with CellTrace™ Violet (CTV) and rested overnight, followed by magnetic enrichment of CMV-specific T cells using Cytokine Secretion Assay and CMV_pp65 stimulation. Afterwards, the T cells were expanded on irradiated autologous PBMCs in presence or absence of indicated immunosuppressants, followed by flow cytometric analysis. **(A)** Gating strategy for analysis of T-cell proliferation. **(B-C)** Bar graph shows summarized mean fluorescent intensities (MFIs) of CTV from the starting material (Day 1) and from proliferating T cells on day 5. Statistical significance (in comparison to UT) was using Friedman test followed by Dunn’s multiple comparison. *p<0.05, **p<0.01, ***p<0.001. UT untreated, SIR/S sirolimus, EVR/E everolimus, TAC/T tacrolimus, MPA/M mycophenolic acid, PRE/P prednisolone.


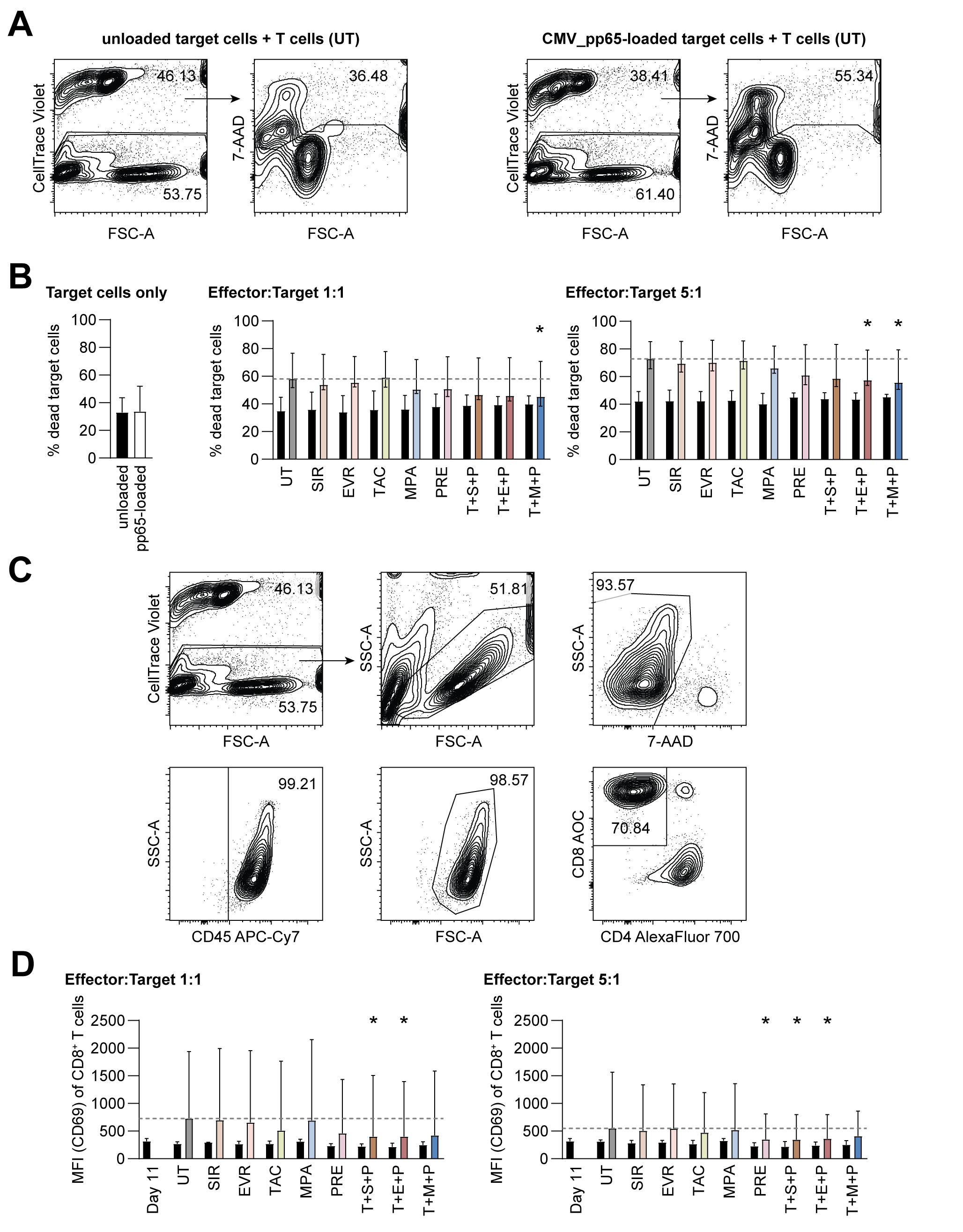


**Figure S8: Functionality of CMV-specific T cells under immunosuppression.** PBMCs were isolated from CMV+ donors and rested overnight, followed by magnetic enrichment of CMV-specific T cells using Cytokine Secretion Assay and CMV_pp65 stimulation. The T cells were expanded on irradiated autologous PBMCs for 11 days and subsequently co-cultured with CTV-labeled autologous CMV_pp65-loaded PBMCs in different effector-to-target ratios and in presence or absence of indicated immunosuppressants. Unloaded PBMCs served as negative control (black bars). After 4h, the frequencies of dead (7-AAD^+^) target cells **(A-B)** and the CD69 expression of CD8+ T cells **(C-D)** were analyzed using flow cytometry. **(A, C)** Gating strategy. **(B, D)** Bar graphs show median and interquartile range Q1-Q3. Statistical significance (in comparison to UT) was calculated using Friedman test followed by Dunn’s multiple comparison (n=4). *p<0.05, **p<0.01, ***p<0.001. UT untreated, SIR/S sirolimus, EVR/E everolimus, TAC/T tacrolimus, MPA/M mycophenolic acid, PRE/P prednisolone.


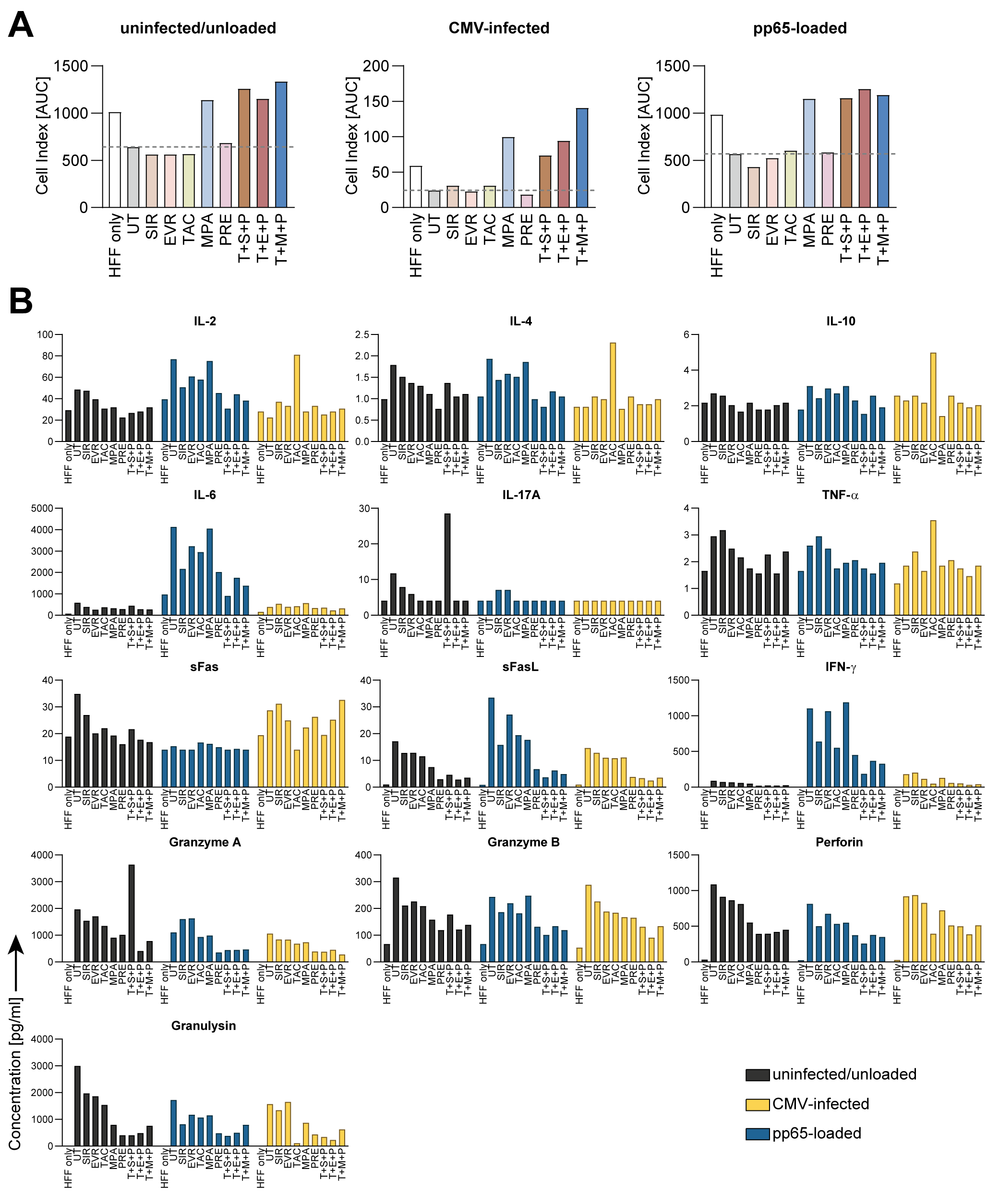


**Figure S9: Cytotoxic capacity of CMV-specific T cells towards CMV-infected fibroblasts under immunosuppression.** PBMCs were isolated from CMV+ donors and rested overnight, followed by magnetic enrichment of CMV-specific T cells using Cytokine Secretion Assay and CMV_pp65. The T cells were expanded on irradiated autologous PBMCs for 11 days and subsequently co-cultured with uninfected, CMV-infected of CMV_pp65-loaded Human Foreskin Fibroblasts (HFF) in an effector-to-target ratio of 1:1 and in presence or absence of indicated immunosuppressants using an xCELLigence RTCA S16 Real Time Cell Analyzer. **(A)** Bar graphs display the AUC of growth curves. **(B)** After seven days, cell culture supernatants were collected and the concentration of cytotoxic molecules was analyzed using a flow cytometry-based multiplex assay (LEGENDplex).

**References**

1. Brunet M, van Gelder T, Asberg A, et al. Therapeutic Drug Monitoring of Tacrolimus-Personalized Therapy: Second Consensus Report. *Ther Drug Monit*. Jun 2019;41(3):261-307. doi:10.1097/FTD.0000000000000640

2. Shipkova M, Hesselink DA, Holt DW, et al. Therapeutic Drug Monitoring of Everolimus: A Consensus Report. *Ther Drug Monit*. Apr 2016;38(2):143-69. doi:10.1097/FTD.0000000000000260

3. MacDonald A, Scarola J, Burke JT, Zimmerman JJ. Clinical pharmacokinetics and therapeutic drug monitoring of sirolimus. *Clin Ther*. 2000;22 Suppl B:B101-121. doi:10.1016/s0149-2918(00)89027-x

4. Bergan S, Brunet M, Hesselink DA, et al. Personalized Therapy for Mycophenolate: Consensus Report by the International Association of Therapeutic Drug Monitoring and Clinical Toxicology. *Ther Drug Monit*. Apr 1 2021;43(2):150-200. doi:10.1097/FTD.0000000000000871

5. Skauby RH, Gustavsen MT, Andersen AM, et al. Prednisolone and Prednisone Pharmacokinetics in Adult Renal Transplant Recipients. *Ther Drug Monit*. Apr 1 2021;43(2):247-255. doi:10.1097/FTD.0000000000000835

6. Qin RR, Qin JW, Li XF, et al. Influence of immunosuppressive drugs on natural killer cells in therapeutic drug exposure in liver transplantation. *Hepatobiliary Surgery and Nutrition*. Feb 27 2023;doi:10.21037/hbsn-22-438

7. Bonifacius A, Tischer-Zimmermann S, Santamorena MM, et al. Rapid Manufacturing of Highly Cytotoxic Clinical-Grade SARS-CoV-2-specific T Cell Products Covering SARS-CoV-2 and Its Variants for Adoptive T Cell Therapy. *Front Bioeng Biotechnol*. 2022;10:867042. doi:10.3389/fbioe.2022.867042

8. Wu Z, Lau CM, Sottile R, et al. Human Cytomegalovirus Infection Promotes Expansion of a Functionally Superior Cytoplasmic CD3(+) NK Cell Subset with a Bcl11b-Regulated T Cell Signature. *J Immunol*. Nov 15 2021;207(10):2534-2544. doi:10.4049/jimmunol.2001319
